# Sulfadiazine selection enables high‐efficiency transformation in *Nannochloropsis* and *Microchloropsis*

**DOI:** 10.1111/tpj.71063

**Published:** 2026-07-31

**Authors:** Jannis Straube, Christopher Robin Wood, Jonna Pohjankukka, Johan Andersen‐Ranberg

**Affiliations:** ^1^ Plant Biochemistry, Department of Plant and Environmental Sciences University of Copenhagen Frederiksberg C 1871 Denmark

**Keywords:** genetic engineering, *Nannochloropsis*, *Microchloropsis*, sulfadiazine, selectable marker, folate, astaxanthin

## Abstract

*Nannochloropsis* and *Microchloropsis* are important as eustigmatophyte model organisms and the production of high‐value lipids. Efficient selectable markers are indispensable for genetic engineering to study and improve production yield of these species. Here, we establish sulfadiazine resistance mediated by the bacterial *sul1* gene as an efficient and robust selection system. Growth assays identified 300 μg·mL^−1^ sulfadiazine as an effective selection concentration. Mitochondrial targeting of Sul results in transformation efficiencies and false‐positive rates superior to commonly used markers aphVII and Sh ble. Sulfadiazine‐resistant transformants exhibit stable growth under selection and no cross‐resistance to hygromycin B, Zeocin, blasticidin S, G418, or nourseothricin. Expression of *Chromochloris zofingiensis β*‐carotene ketolase using the Sul selectable marker in *Nannochloropsis oceanica* and *Microchloropsis gaditana* resulted in accumulation of the ketocarotenoids canthaxanthin and adonirubin, producing a distinct brown phenotype. Mitochondrial‐targeted Sul thus provides a robust, high‐efficiency selectable marker for transformation and genetic engineering in *Nannochloropsis* and *Microchloropsis*.

## Significance Statement

Genetic engineering in the marine eustigmatophytes *Nannochloropsis* and *Microchloropsis* is constrained by intrinsic antibiotic resistance and reduced marker efficacy at high salinity. We show that mitochondrial‐targeted bacterial Sul provides a robust, cost‐effective selectable marker, enabling sulfadiazine selection with minimal background. This system supports stable transformant growth, avoids cross‐resistance, and is suitable for stable gene expression.

## INTRODUCTION

*Nannochloropsis* and *Microchloropsis* are marine eustigmatophyte microalgae (Fawley et al., 2015) with considerable potential as photosynthetic biomanufacturing host organisms due to their rapid growth, high biomass productivity, and ability to accumulate proteins (Williamson et al., 2024) and high levels of high‐value lipids and other value‐added metabolites. *Nannochloropsis* and Microchloropsis species can store up to 60% of their dry weight as lipids (Morales et al., 2021), including polyunsaturated fatty acids (PUFAs), such as eicosapentaenoic acid (EPA) (Poliner, Pulman, et al., 2018). Both genera also produce high levels of carotenoids, notably canthaxanthin and astaxanthin, compartmentalized in the red body (Gee et al., 2024), which are widely exploited in food, feed, and aquaculture. Beyond their biomanufacturing potential, marine eustigmatophytes contribute to the oceanic carbon cycle through long‐term carbon storage in their robust, aliphatic algaenan cell wall, which is highly recalcitrant and resistant to degradation, contributing to sedimentary carbon sequestration (Rampen et al., 2012), an aspect of growing importance in the context of climate change. Thus, *Nannochloropsis* and *Microchloropsis* are model organisms for eugstigmatophyte algae and genomic and molecular biology tools have greatly advanced our understanding of biology and metabolism underlying these traits, although significant gaps remain.

The first *Nannochloropsis* genome was sequenced by Vieler et al. (2012). Since then, numerous nuclear, mitochondrial, and chloroplast genomes from *Nannochloropsis* and *Microchloropsis* species, along with transcriptomes, have been sequenced and annotated (Corteggiani Carpinelli et al., 2014; Radakovits et al., 2012; Wang et al., 2014; Wei et al., 2013), establishing the foundation for robust genetic engineering. Building on these resources, a range of molecular tools has been developed for *Nannochloropsis* and *Microchloropsis*, including episomal vectors, CRISPR‐Cas9 and Cas12a systems (Naduthodi et al., 2019; Poliner et al., 2020; Poliner, Farré, & Benning, 2018; Poliner, Takeuchi, et al., 2018; Wang et al., 2016), TALENs (Kurita et al., 2020), and CRISPR‐Cas9‐mediated base editors (Moroi et al., 2025).

Efficient and reliable genetic engineering depends on the availability of selectable markers. To date, six markers have been established for *Nannochloropsis* and *Microchloropsis* species, enabling selection of transformants with genetically engineered traits. These include aminoglycoside phosphotransferase (aphVII) conferring hygromycin B resistance (Vieler et al., 2012); bleomycin resistance protein (Sh ble) conferring Zeocin resistance (Poliner, Pulman, et al., 2018); blasticidin S deaminase (bsd) conferring blasticidin S resistance; neomycin‐3′‐O‐phosphotransferase (NeoR) conferring geneticin (G418) resistance; nourseothricin acetyltransferase (NAT) conferring nourseothricin resistance (Poliner et al., 2020); and a mutated phytoene desaturase derived from *Nannochloropsis oceanica* conferring resistance to norflurazon (Liu et al., 2022). Among these, selection systems based on hygromycin B and Zeocin are the most used in *Nannochloropsis* and *Microchloropsis* species (Table S1). However, Zeocin causes random DNA double‐strand breaks and is mutagenic (Lin et al., 2017), which may limit its utility. Moreover, established selection schemes, including those based on hygromycin B and G418, exhibit reduced efficacy under high‐salinity conditions, posing a significant challenge for biomanufacturing applications involving marine algal species (Takahashi et al., 2011). Expansion of the selectable marker repertoire remains a challenge, as marine eustigmatophytes exhibit intrinsic resistance to many commonly used selective agents, including rifampicin, benomyl, nystatin, spectinomycin, ampicillin, and chloramphenicol (Vieler et al., 2012).

**Figure 1:**
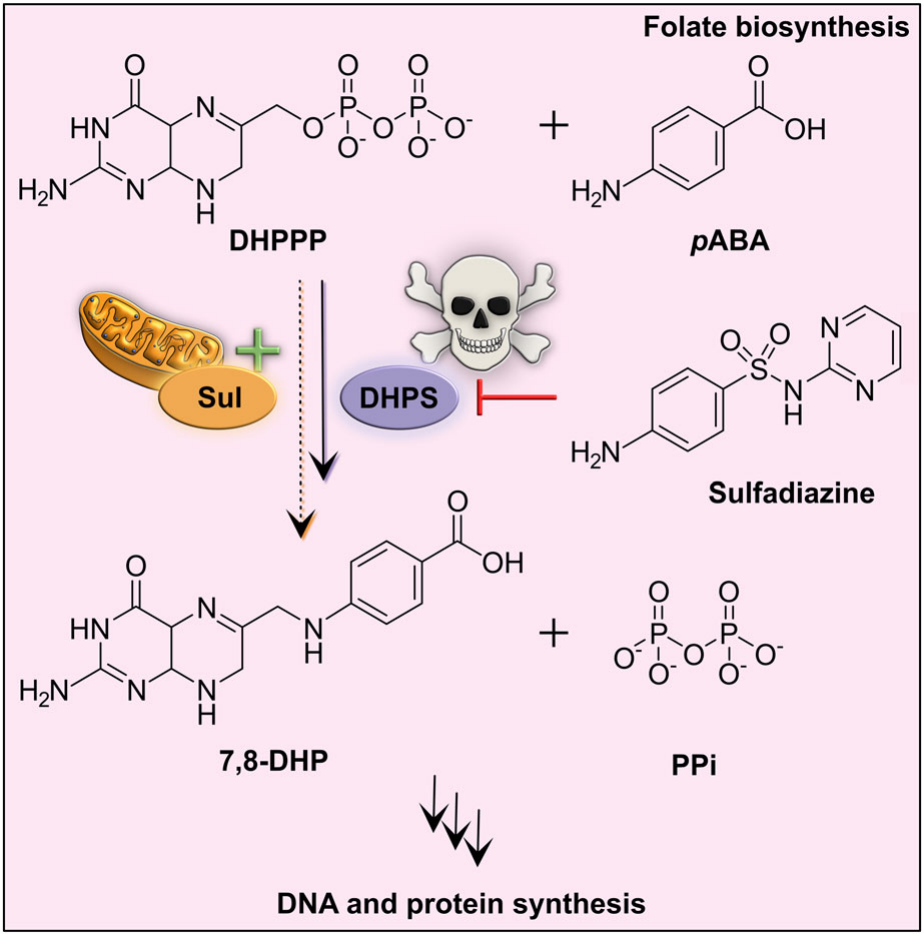
Schematic illustration of the sulfadiazine‐based selection mechanism. Sulfadiazine inhibits mitochondrial dihydropteroate synthase (DHPS), the enzyme that catalyzes the conversion of dihydropteridine pyrophosphate (DHPPP) and *p*‐aminobenzoic acid (*p*ABA) into 7,8‐dihydropteroate (7,8‐DHP) and pyrophosphate (PPi) in the folate biosynthesis pathway. Expression of the bacterial *sul1* gene, which encodes a sulfadiazine‐resistant DHPS (Sul), restores this pathway.

Sulfonamides, including sulfadiazine, represent promising selectable agents by inhibiting dihydropteroate synthase (DHPS), a key enzyme in the folate pathway (Venkatesan et al., 2023). The pathway's product, tetrahydrofolate, serves as a one‐carbon carrier and an essential cofactor for core metabolism, including the biosynthesis of nucleotides and amino acids (Hanson & Gregory, 2002, 2011). Consequently, disruption of the folate pathway is lethal. In *Enterobacteriaceae*, a sulfadiazine‐resistant variant of DHPS (Sul), encoded by the *sul1* gene, restores folate biosynthesis in the presence of sulfonamides (Guerineau, Brooks, Meadows, et al., 1990; Guerineau, Brooks, & Mullineaux, 1990) (Figure 1). Leveraging this principle, the *sul1* gene has been successfully used as a selectable marker in plants (Guerineau, Brooks, Meadows, et al., 1990; Hadi et al., 2002; Robinson et al., 2024; Tabatabaei et al., 2019; Thomson et al., 2011; Wallis et al., 1996) and in the freshwater algae *Chlamydomonas reinhardtii* (Tabatabaei et al., 2019) and *Cyanidioschyzon merolae* (Borges‐Rodríguez et al., 2026). While most plant studies target Sul to the chloroplast, the final five steps of the folate biosynthesis, including DHPS, are localized in the mitochondria (Hanson & Gregory, 2011). Consistent with this localization, Tabatabaei et al. (2019) and Borges‐Rodríguez et al. (2026) demonstrated that mitochondrial targeting of Sul yields the highest efficiency in selecting sulfadiazine‐resistant transformants.

The Sul marker selection system has been developed for plants and freshwater algae; however, it has not been adapted for marine algae. In this study, we address this gap by determining the lethal dose of sulfadiazine in marine eustigmatophytes, establishing the Sul marker for *Nannochloropsis* and *Microchloropsis*, and demonstrating sulfadiazine selection for stable gene expression.

## RESULTS

### Determination of lethal dose of sulfadiazine in marine eustigmatophytes

The sensitivity of *Nannochloropsis* spp. and *Microchloropsis* spp. to sulfonamides was established by exposing cells to increasing concentrations of sulfadiazine on agar selection plates (Table 1, Figure S1). At low sulfadiazine concentrations (100 and 200 μg·mL^−1^), a limited number of colonies were formed in *Nannochloropsis oceanica* CCMP1779, *Nannochloropsis oceanica* CCAP 211/78, and *Microchloropsis gaditana*, whereas *Microchloropsis salina* exhibited reduced sensitivity. Exposure to higher concentrations (≥300 μg·mL^−1^) resulted in complete growth inhibition and cell death for all four species. A similar response was observed in liquid cultures of *N. oceanica* CCMP1779 and *M. gaditana* (Figure S2). While 300 μg·mL^−1^ can work for selection of transformants, 500 μg·mL^−1^ was chosen as a suitable sulfadiazine concentration and used as the standard concentration in subsequent experiments to ensure efficient elimination of false‐positive transformants.

**Table 1:** Number of colonies formed by *Nannochloropsis* and *Microchloropsis* species grown for 4 weeks on solid f/2 N media containing varying concentrations of sulfadiazine. Data are presented as means ± SD (*n* = 4)

Species	Strain	Sulfadiazine (μg·mL^−1^)	Number of colonies
*Nannochloropsis oceanica*	CCMP1779	0	Full patch
		100	9.8 ± 4.4
		200	0 ± 0
		300	0 ± 0
		400	0 ± 0
		500	0 ± 0
		1000	0 ± 0
*Nannochloropsis oceanica*	CCAP 211/78	0	Full patch
		100	0 ± 0
		200	0 ± 0
		300	0 ± 0
		400	0 ± 0
		500	0 ± 0
		1000	0 ± 0
*Microchloropsis gaditana*	CCAP 849/6	0	Full patch
		100	23.8 ± 38.8
		200	2.3 ± 3.9
		300	0 ± 0
		400	0 ± 0
		500	0 ± 0
		1000	0 ± 0
*Microchloropsis salina*	CCAP 849/3	0	Full patch
		100	4744.0^a^ ± 1409.8
		200	0.3 ± 0.4
		300	0 ± 0
		400	0 ± 0
		500	0.3 ± 0.4
		1000	0 ± 0

^a^ Extrapolated from ¼ plate due to high colony count.

### Establishment of Sul resistance marker in *Nannochloropsis* and *Microchloropsis*

Following confirmation of sulfadiazine susceptibility, we generated constructs expressing *sul1* variants targeted to different cellular compartments, including a chloroplast‐targeted version (cTP), a long mitochondrial targeting peptide (lMTP), and its truncated form (sMTP), and a cytosolic version, all under the moderately active Ribi promoter (pRibi) (Poliner, Pulman, et al., 2018). For comparison, constructs carrying the Zeocin resistance gene (*Sh ble*) and hygromycin B resistance (*aphVII*) were generated (Figure S3).

**Figure 2:**
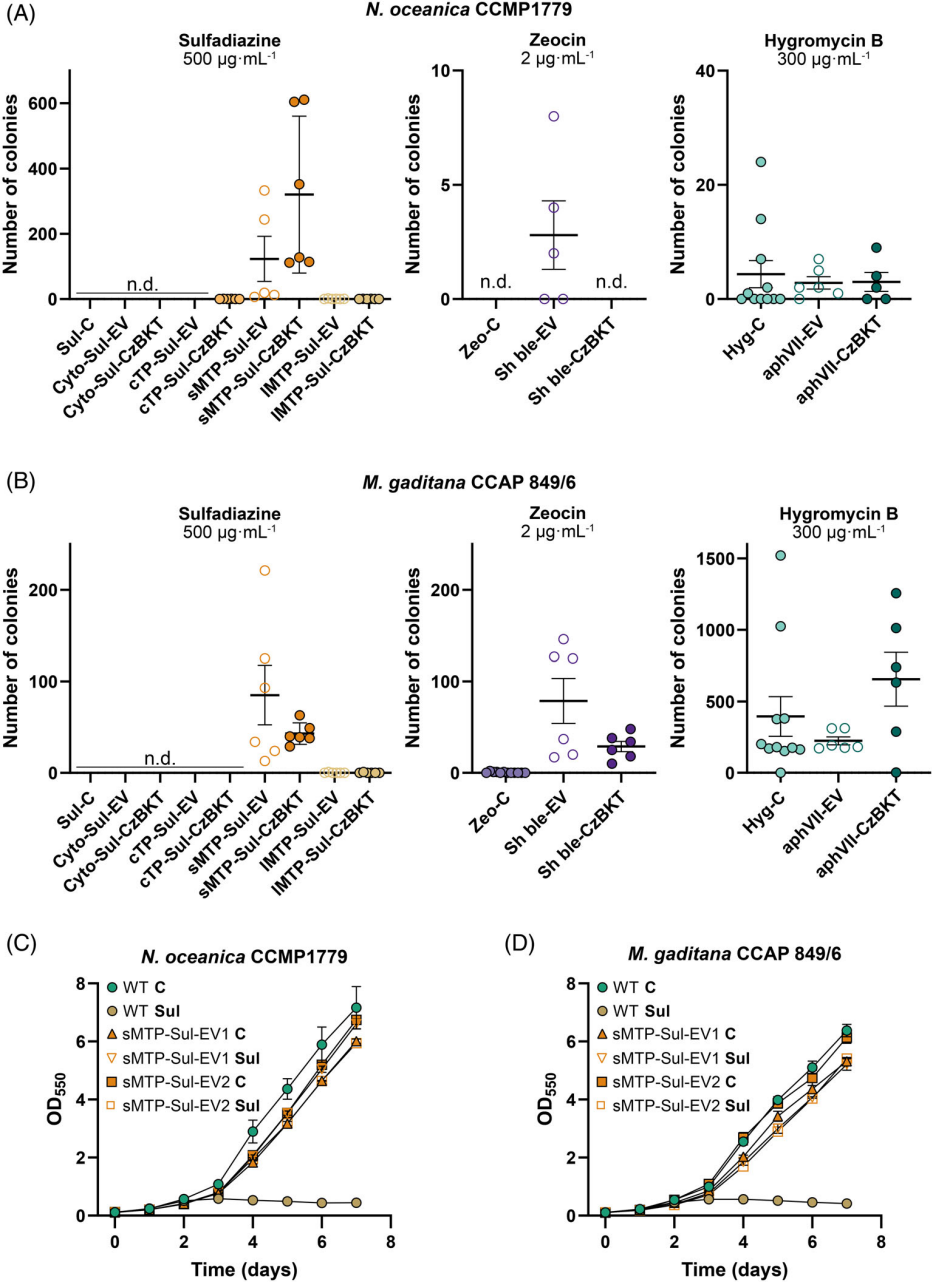
Heterologous *sul1* expression confers resistance to sulfadiazine in *Nannochloropsis oceanica* and *Microchloropsis gaditana*. Colony counts indicating transformation efficiency with aphVII, Sh ble, and Sul selectable markers, with Sul targeted to the cytoplasm (Cyto), chloroplast (cTP), or mitochondria via short or long mitochondrial targeting peptides (sMTP, lMTP) in (A) *N*. *oceanica* (*n* = 4–6) and (B) *M. gaditana* (*n* = 5–6). Selective agent‐specific controls (Sul‐C, Zeo‐C, Hyg‐C) were included for each species (*n* = 11–12). Growth of (C) *N*. *oceanica* and (D) *M*. *gaditana* wild‐type (WT) and two independent sMTP‐Sul‐EV lines in f/2 N media, with 500 μg·mL^−1^ sulfadiazine (Sul) or without (C), was monitored every 24 h for 7 days by measuring optical density at 550 nm (*n* = 3). Data are presented as mean ± SD. nd, not detected.

Transformation efficiency was evaluated using empty vector controls (EV; expressing mScarlet‐I3 only) and constructs containing the chloroplast‐targeted *Chromochloris zofingiensis ß*‐carotene ketolase (CzBKT) (Gee et al., 2024; Roth et al., 2017). Only transformants expressing mitochondria‐targeted Sul were recovered on sulfadiazine selection plates. Cytosolic constructs produced no colonies, and chloroplast‐targeted constructs resulted in only two colonies across six replicates in *N. oceanica*. This demonstrates that mitochondrial localization is required for Sul‐mediated selection on sulfadiazine selection plates. Notably, sMTP‐Sul constructs generated substantially more transformants than lMTP‐Sul constructs (Figure 2A,B).

Consistently with this trend, mitochondrially targeted Sul produced the highest number of transformants for *N*. *oceanica* and *M*. *gaditana*, while no colonies were observed on control plates (Figure 2A,B; Figures S4 and S5). In *M. gaditana*, sMTP‐Sul (EV: 85.0 ± 79.6; CzBKT: 43.0 ± 11.7) and Sh ble (EV: 78.7 ± 60.0; CzBKT: 28.8 ± 13.9) constructs produced comparable colony numbers. In contrast, in *N. oceanica*, sMTP‐Sul constructs (EV: 123.2 ± 154.2; CzBKT: 320.2 ± 240.4) generated substantially more transformants than Sh ble constructs (EV: 2.8 ± 3.3; CzBKT 0.0 ± 0.0). Selection with aphVII (EV: 223.8 ± 68.7; CzBKT: 655.0 ± 459.9) on hygromycin B selection plates yielded the highest colony numbers in *M. gaditana*, however, a significant number of colonies was observed for control strains of both species plated on 300 μg·ml^−1^ hygromycin B (Figure 2A,B; Figures S4 and S5).

sMTP‐Sul‐EV transformants of *N*. *oceanica* and *M*. *gaditana* exhibited growth on 500 μg mL^−1^ sulfadiazine that was comparable to their growth under control conditions in f/2 N medium. No growth was observed for wild‐type (WT) cultures, confirming the effectiveness of the Sul‐based selection system (Figure 2C,D).

**Figure 3:**
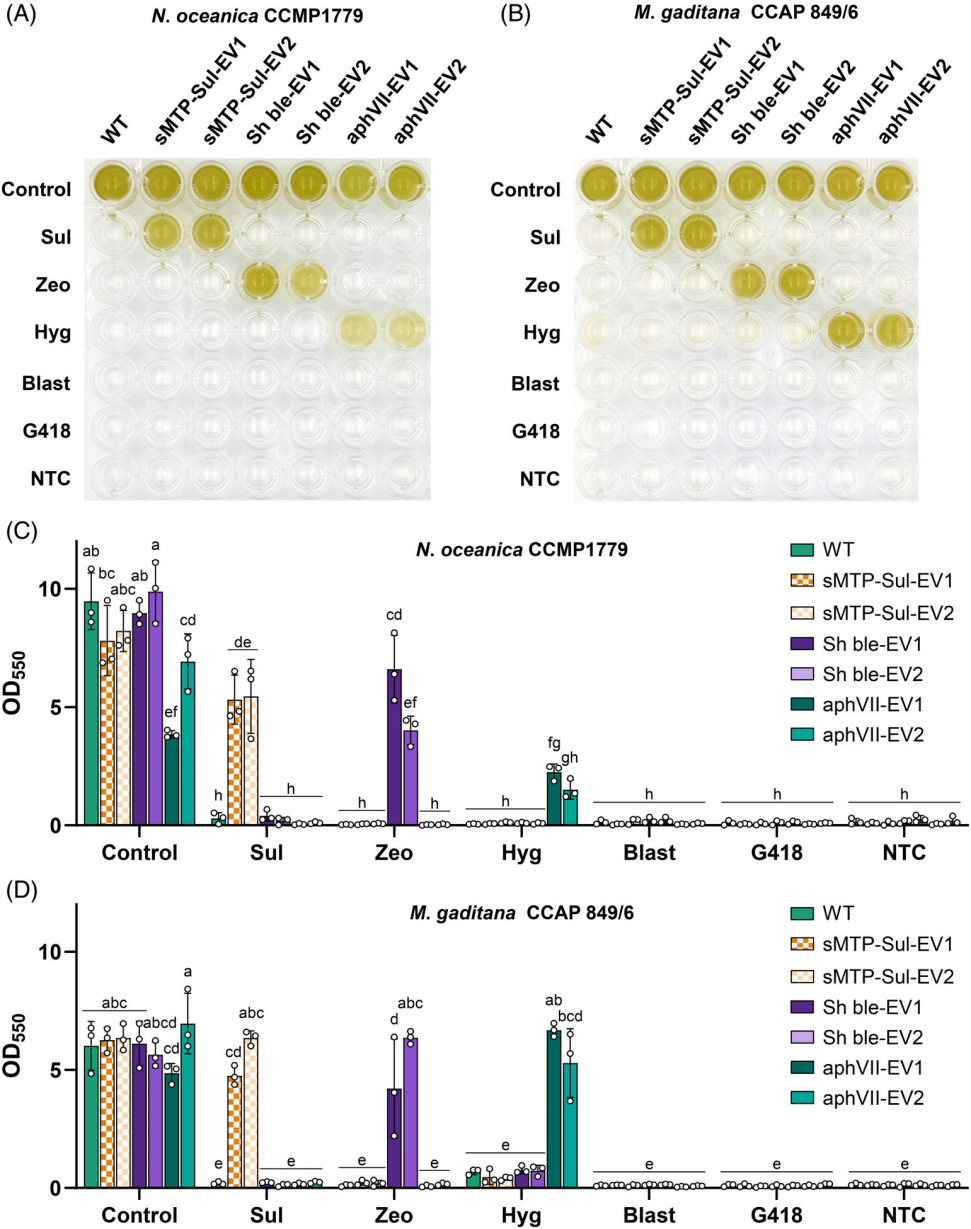
Sulfadiazine‐resistant colonies remain sensitive to commonly used selective agents. (A) *N*. *oceanica* and (B) *M. gaditana* wild‐type (WT), two independent sMTP‐Sul‐EV lines, two independent Sh ble‐EV lines, and aphVII‐EV lines were assessed for cross‐resistance to commonly used selective agents in these species. Cultures were grown in f/2 N medium without selective agent (Control) or supplemented with sulfadiazine (Sul, 500 μg·mL^−1^), Zeocin (Zeo, 2 μg·mL^−1^), hygromycin B (Hyg, 300 μg·mL^−1^), blasticidin S (Blast, 25 μg·mL^−1^), G418 (250 μg·mL^−1^), or nourseothricin (NTC, 200 μg·mL^−1^) for 7 days. Growth was assessed in (C) *N*. *oceanica* and (D) *M*. *gaditana* by measuring optical density at 550 nm. Data are presented as mean ± SD (*n* = 3). Different letters indicate significant differences (two‐way ANOVA, Tukey's HSD, *P* ≤ 0.05).

We assessed whether expression of Sul confers resistance to other commonly used selectable agents in *N. oceanica* and *M. gaditana*, including Zeocin, hygromycin B, blasticidin, G418, and nourseothricin. sMTP‐Sul‐EV lines grew only in f/2 N medium with or without sulfadiazine and showed no growth on any other tested antibiotics, indicating the absence of cross‐resistance (Figure 3A–D). Similarly, Sh ble‐EV lines grew only with or without Zeocin, and aphVII‐EV lines only with or without hygromycin B, with no cross‐resistance to sulfadiazine or other agents (Figure 3A–D). Notably, some growth was observed in *M. gaditana* grown in f/2 N containing hygromycin B, albeit at lower levels than in the control, consistent with previously observed background growth on plates (Figure 3B,D), indicating limited usefulness of hygromycin B as a selective agent at 300 μg mL^−1^.

### Stable expression of CzBKT under sulfadiazine selection

**Figure 4:**
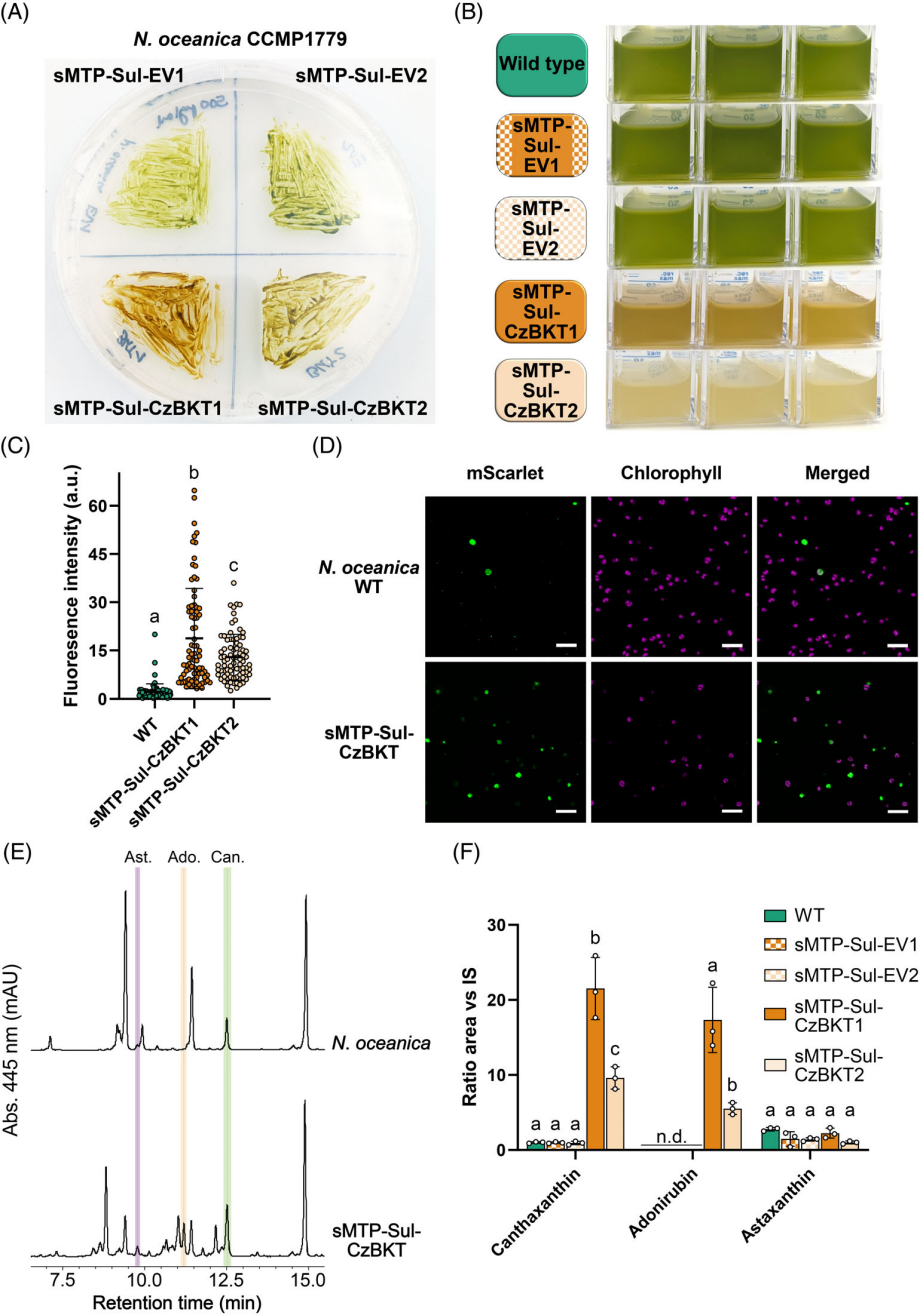
*CzBKT* expression in sulfadiazine‐resistant *Nannochloropsis oceanica* leads to accumulation of ketocarotenoids. *N. oceanica* harboring the sMTP‐Sul‐CzBKT construct exhibited a pronounced brown phenotype compared with the wild‐type (WT) strain and empty vector control (sMTP‐Sul‐EV). (A) Representative colonies grown on agar plates containing 500 μg·mL^−1^ sulfadiazine, showing strains harboring either the sMTP‐Sul‐EV or sMTP‐Sul‐CzBKT construct. (B) Liquid cultures in f/2 N medium grown in triplicate, including wild‐type controls and transformant lines derived from plate selection. sMTP‐Sul‐CzBKT lines consistently showed a brown phenotype relative to WT and EV controls. Chloroplast‐targeted CzBKT expression was evaluated by single‐cell quantification of mScarlet‐I3 fluorescence intensity in (C, D) *N. oceanica* wild‐type (WT) and sMTP‐Sul‐CzBKT‐expressing lines. Confocal images show chlorophyll autofluorescence (Ex: 568 nm, Em: 676–762 nm) and mScarlet‐I3 (Ex: 568 nm, Em: 578‐640 nm), along with merged channels. The white scale bars represent 10 μm. Fluorescence intensity was analyzed by one‐way ANOVA followed by Tukey's HSD test. Different letters indicate significant differences (*P* ≤ 0.05). Data are shown as mean ± SD with individual data points. (E) *N*. *oceanica* expressing sMTP‐Sul‐CzBKT lines display new carotenoid peaks detected by UV absorbance at 445 nm and confirmed by mass spectrometry, compared with the WT. Peaks corresponding to canthaxanthin (Can., green), adonirubin (Ado., yellow), and astaxanthin (Ast., purple) are highlighted in the chromatogram (*n* = 3). (F) Relative quantification from extracted ion chromatogram (EICs; ±0.01 m/z window) peak area ratios to the internal standard (10 ppm 8‐apo‐carotenal; m/z 417.3152 [M + H]^+^) of canthaxanthin (m/z 565.4040 [M + H]^+^), adonirubin (m/z 581.3989 [M + H]^+^) and astaxanthin (m/z 597.3989 [M + H]^+^) in two independent sMTP‐Sul‐CzBKT lines, two independent sMTP‐Sul‐EV lines and the WT in *N*. *oceanica*. Data are presented as mean ± SD (*n* = 3). Different letters indicate statistically significant differences among groups for each metabolite, as determined by one‐way ANOVA followed by Tukey's HSD test (*P* ≤ 0.05). n.d., not detected.

*N*. *oceanica* and *M*. *gaditana* sMTP‐Sul‐CzBKT transformants were investigated for their potential to produce valuable ketocarotenoids via overexpression of *CzBKT* (Gee et al., 2024; Roth et al., 2017) fused to the red fluorescent protein mScarlet‐I3. Transformants exhibited a distinct brown phenotype on agar plates and in liquid culture compared with sMTP‐Sul‐EV transformants and WT (Figure 4A,B; Figure S6a,b). CzBKT expression was confirmed by confocal microscopy showing a strong mScarlet signal (Figure 4C,D; Figure S6c,d). Notably, red bodies in sMTP‐Sul‐CzBKT lines appeared larger than in WT and exhibited strong autofluorescence, consistent with previous reports (Gee et al., 2024). A weak signal was detected in the mScarlet channel in control samples, primarily attributable to red body autofluorescence (Figure 4C,D; Figure S6c,d). Pigment analysis revealed that CzBKT‐expressing lines accumulated the ketocarotenoids canthaxanthin and adonirubin, that are only found in trace amounts in WT. Astaxanthin levels remained largely unchanged in the CzBKT‐expressing lines compared with WT (Figure 4E,F; Figure S6e,f), demonstrating that sulfadiazine selection effectively enables heterologous protein expression in *N*. *oceanica* and *M*. *gaditana*.

## DISCUSSION

Here, we demonstrate that sulfadiazine‐based selection using the bacterial *sul1* gene, encoding Sul and fused to an engineered mitochondrial targeting peptide, enables efficient selection in *Nannochloropsis* and *Microchloropsis* species, with efficiency comparable to or exceeding that of established selectable markers. Across four species, effective selection was achieved at 300 μg·ml^−1^ sulfadiazine. At 100 μg·ml^−1^, colony formation varied between species, with some species showing substantial growth and others fewer colonies. In plants, Sul‐based selection is achieved with 1 μg·ml^−1^ in *Marchantia polymorpha* (Robinson et al., 2024), 5 μg·ml^−1^ in *Arabdiopsis thaliana* (Hadi et al., 2002), and 25 μg·ml^−1^ in *Nicotiana tabacum* and *Solanum tuberosum* (Tabatabaei et al., 2019; Wallis et al., 1996). Similarly, the freshwater alga *C. merolae* shows low tolerance, with 5 μg·mL^−1^ sufficient for selection (Borges‐Rodríguez et al., 2026). *Nannochloropsis* and *Microchloropsis* selection was achieved with sulfadiazine concentrations used for selection of cell wall‐deficient *C. reinhardtii* transformants with a minimum inhibitory concentration of 500 μg·ml^−1^. However, our results indicate that selection may also be feasible at lower concentrations, potentially as low as 300 μg·ml^−1^, as suggested by our dose–response analysis. In contrast, *C*. *reinhardtii* strains with intact cell walls exhibited even higher tolerance, requiring up to 1200 μg·ml^−1^ for growth inhibition (Tabatabaei et al., 2019).

In plants, strong promoters such as Cauliflower mosaic virus 35S promoter permit selection with chloroplast‐targeted Sul (Robinson et al., 2024; Wallis et al., 1996), whereas weaker promoters allow selection only when Sul is targeted to the mitochondria (Tabatabaei et al., 2019). Mitochondrial targeting of Sul profoundly affects selection efficiency in marine eustigmatophytes. Among the homologs of *A. thaliana* dihydropteroate synthase (AtDHPS) identified in eustigmatophytes genome models, most were predicted to contain a mitochondrial targeting signal, providing a plausible explanation for the strict requirement for mitochondrial targeting of the Sul enzyme in the sulfadiazine‐based selection system (Table S2). A shortened version of the established mitochondrial targeting peptide derived from *oxidase assembly protein 1* (*NoOXA1*) (Moog et al., 2015) was used to infer sulfadiazine selection. Enhanced mitochondrial targeting of the truncated sMTP in *N*. *oceanica* and *M*. *gaditana* can be attributed to its increased electrostatic compatibility with the mitochondrial import machinery. At physiological pH (7.0), removal of acidic residues (aspartic and glutamic acid) from the lMTP raises the net positive charge of the sMTP (+14.4*e*) relative to the lMTP (+10.6*e*). Concomitantly, truncation nearly doubles the charge density (+0.19 versus +0.10 per residue), thereby strengthening the electrostatic potential of the targeting sequence. Given that mitochondrial import via the translocase of the outer membrane (TOM) and inner membrane (TIM) complexes relies on the recognition of positively charged, amphipathic *α*‐helices, these changes are expected to enhance both binding to the mitochondrial surface and subsequent translocation efficiency (Schmidt et al., 2010).

We further demonstrated that sulfadiazine‐based selection is suitable for heterologous expression of CzBKT. Sulfadiazine‐resistant transformants carrying the *CzBKT* gene accumulated canthaxanthin and adonirubin, while astaxanthin levels remained unchanged. This observation is consistent with previous reports in *Nannochloropsis* expressing CzBKT. In contrast, Liu et al. (2024) reported a substantial increase in astaxanthin upon expression of *Haematococcus pluvialis* CHYB, an enzyme with high affinity for hydroxylation at the 3‐position of the *β*‐ionone rings. In *Nannochloropsis*, this hydroxylation step is hypothesized to be catalyzed by NoCYP97F5 (Gee et al., 2024; Leonelli et al., 2016), however, insufficient activity on ketolated beta‐carotenoid substrates could limit conversion to astaxanthin.

The use of *Nannochloropsis* or *Microchloropsis* species as hosts for metabolic engineering for photosynthetic production of high‐value compounds will likely require the integration of multiple genes for the reconstitution of complex metabolic pathways. In this context, sulfadiazine resistance is well suited for gene‐stacking approaches, as it shows no cross‐resistance with any of the five commonly used selective agents. Still, transformation efficiency decreases with increasing construct size in a species‐specific manner (Hanahan, 1983; Sheng et al., 1995), and the use of multiple selectable antibiotics may further reduce production yields of high‐value compounds (Palanisamy et al., 2019), as demonstrated in *Escherichia coli*. A recent study circumvented this limitation by demonstrating intein‐mediated splicing of aphVII and Sul in plants, enabling the simultaneous selection of multiple integrated expression cassettes using a single selective agent (Moratti et al., 2025). By establishing the Sul selection marker in this study, this approach can be employed to streamline complex metabolic engineering in marine eustigmatophytes.

## CONCLUSION

Our results demonstrate that mitochondrially targeted Sul is an effective selection marker for genetic transformation in *Nannochloropsis* and *Microchloropsis* species. Compared with commonly used antibiotic resistance markers such as aphVII and Sh ble, the Sul selection system produces higher and more consistent transformation efficiencies, thereby providing an improved tool for genetic engineering and functional studies in marine eustigmatophytes.

## MATERIALS AND METHODS

### Culture conditions and sulfadiazine dose–response assay

*Nannochloropsis* and *Microchloropsis* species were grown photoautotrophically in f/2 N medium (210 mM NaCl, 4.7 mM KCl, 4.2 mM KBr, 0.36 mM NaF, 2.43 mM H_3_BO_3_, 14.40 mM Na_2_SO_4_, 11.9 mM NaHCO_3_, 0.03 mM SrCl_2_*6H_2_0, 27.3 mM MgCl_2_*6H_2_O, 5.24 mM CaCl_2_*2H_2_O, 10 mM Tris–HCl (pH 7.6), 0.36 mM NaH_2_PO_4_*H_2_O, 0.19 mM Na_2_CO_3_, 24 μM FeCl_3_*6H_2_O, 125 μM Na_2_EDTA‐HCl (pH 8), 0.2 μM CuSO_4_*5H_2_O, 0.13 μM Na_2_MoO_4_, 0.38 μM, ZnSO_4_*7H_2_O, 0.24 μM CoCl_2_*6H_2_O, 4.5 μM MnCl_2_*4H_2_O, 20.5 nM biotin, 3.7 nM vitamin B_12_, 2.96 μM Thiamin HCl) using 5 mM KNO_3_ as the sole nitrogen source. Liquid cultures were cultivated in an Algaetron AG230 (Photo Systems Instruments, Drásov, Czech Republic) at 26°C under a 12 h light/12 h dark cycle for transformations or under continuous illumination for all other experimental conditions, with light intensity of 80 μmol photons m^−2^ s^−1^, 2% CO_2_ supplementation, and continuous orbital shaking at 142 rpm. Solid cultures were grown on f/2 N agar (0.9% micro agar; Duchefa Biochemie B.V, Haarlem, Netherlands) and incubated at 26°C under continuous illumination (80 μmol photons m^−2^ s^−1^) with 0.2% CO_2_ supplementation. Microalgae species used in this study were *N. oceanica* CCMP1779 (National Center for Marine Algae and Microbiota, Bigelow Laboratory for Ocean Sciences, ME, USA), *N. oceanica* CCAP 211/78 (previously *Nannochloropsis* sp., but reclassified as *N*. *oceanica* by Fawley et al. (2015)), *M. gaditana* CCAP849/6 and *M. salina* CCAP 849/3 (Culture Collection of Algae and Protozoa, SAMS Limited, Dunbeg, Scotland).

To determine the lethal dose of sulfadiazine for *Nannochloropsis* and *Microchloropsis* species, 50 mL of exponentially growing liquid cultures (OD_550_ ≈ 0.4) were pelleted and resuspended before being plated onto solid f/2 N agar supplemented with incrementing concentrations of sulfadiazine sodium salt (0, 100, 200, 300, 400, 500, 1000 μg·ml^−1^; Sigma‐Aldrich, St. Louis, MO, USA; Cat. No. S6387). Colony numbers were counted after 4 weeks of incubation to assess the effects of sulfadiazine on cell growth and viability. For *M. salina* CCAP 849/3 grown at 100 μg·ml^−1^ sulfadiazine, only one quarter of the plate was counted, and the total number of colonies was extrapolated by multiplying by four due to the high colony density.

Transformant regeneration was assessed on selective solid f/2 N agar containing the appropriate antibiotic for each construct: 500 μg·mL^−1^ sulfadiazine, 300 μg·mL^−1^ hygromycin B (Thermo Fisher Scientific, Waltham, MA, USA), or 2 μg·mL^−1^ Zeocin (Thermo Fisher Scientific, Waltham, MA, USA).

### Cloning of expression constructs

The *sul1* sequence was derived from the vector pMpGWBs00 (Robinson et al., 2024) and synthesized as a gene fragment by Twist Bioscience (South San Francisco, CA, USA). Gene fragments were PCR‐amplified using the Q5® High‐Fidelity 2× Master Mix (New England Biolabs, Ipswich, MA, USA) and gene‐specific primers (Table S3; final concentration 500 nM) following the manufacturer's instructions. PCR products were gel purified using the MicroElute® Gel Extraction Kit (Omega Bio‐tek Inc, Norcross, GA, USA). Gene fragments were cloned into the pABNE‐Zeov1 vector using the NEBuilder® HiFi DNA Assembly Master Mix (New England Biolabs, Ipswich, MA, USA) according to the manufacturer's protocol. Expression of both the resistance gene cassette and the gene of interest was driven by the intergenic region between two ribosomal subunit genes (pRibi), which function as a bidirectional promoter (Poliner, Pulman, et al., 2018). Resistance genes (*sul1* with different N‐terminal targeting sequences and *aphVII*; Table S4) were inserted into *SpeI‐HF*‐digested (New England Biolabs, Ipswich, MA, USA) pABNE‐Zeov1 to replace *Sh ble*. *CzBKT* from *C. zofingiensis* (Roth et al., 2017) was cloned into *HindIII*‐digested (New England Biolabs, Ipswich, MA, USA) pABNE‐Zeov1, pABNE‐Hygv1, and pABNE‐Sulv1‐4 expression vectors as a fusion with a C‐terminal mScarlet‐I3 fluorescent protein via a (GSA)_3_ linker (Figure S3). Downstream of the resistance cassettes, a CaMV 35S terminator (T_35S_) was used (Poliner, Takeuchi, et al., 2018), whereas the *mScarlet‐I3* or *CzBKT*‐*mScarlet‐I3* fusion were terminated with the *N*. *oceanica α*‐tubulin terminator (T_*α*‐tub_) (Südfeld, Pozo‐Rodríguez, et al., 2022). Complete plasmid sequences for all constructs described above are provided in Data S1.

### Microalgae transformation

Transformation of *N. oceanica* and *M*. *gaditana* was performed as described by Poveda‐Huertes et al. (2023) with minor modifications. Fifty milliliters of culture at exponential growth (OD_550_ ranging from 0.3 to 0.6) were harvested 6 h after entering the dark phase of the diurnal cycle to maximize transformation efficiency. Cells were washed five times with ice‐cold D‐sorbitol (0.384 mM) and resuspended in 200 μL ice‐cold D‐sorbitol. The suspension was transferred to 2 mm electroporation cuvettes, and 0.5 μg of proofreading PCR‐linearized plasmid DNA was added on ice. Electroporation was carried out using a Gene Pulser Xcell™ Electroporation Total System (Bio‐Rad, Hercules, CA, USA) set to exponential decay mode, with a field strength of 2.2 kV, capacitance of 50 μF, and resistance of 500 Ω. The time constant for the pulse ranged from 22 to 25 ms (Data S2). Immediately after electroporation, cells were transferred to 5 mL of f/2 N medium and incubated overnight in the dark. The following day, cultures were spread onto freshly prepared f/2 N agar containing the appropriate selective antibiotic. Transformant colonies appeared after 3–4 weeks of incubation and were counted after 5 weeks. Colonies were subsequently screened by colony PCR to confirm successful transformation events.

### Growth curves and cross‐resistance test

Twenty milliliters of WT *N. oceanica* and *M. gaditana*, along with two sMTP‐Sul‐EV lines for each strain, were cultivated at an initial OD_550_ of 0.1. Cultures were grown in f/2 N medium either without supplementation or supplemented with 500 μg·mL^−1^ sulfadiazine. Cell growth was monitored at 24 h intervals by measuring the optical density at 550 nm.

For the evaluation of cross‐resistance between selectable markers and selective agents, *N*. *oceanica* and *M*. *gaditana* WT, two independent sMTP‐Sul‐EV lines, two independent Sh ble‐EV lines, and two independent aphVII‐EV lines were tested against commonly used selective agents. Cultures were inoculated at an initial OD_550_ of 0.1 in 96‐well plates and grown in f/2 N medium without selective agent or supplemented with sulfadiazine (500 μg·mL^−1^), Zeocin (2 μg·mL^−1^), hygromycin B (300 μg·mL^−1^), blasticidin S (25 μg·mL^−1^), G418 (250 μg·mL^−1^), or nourseothricin (200 μg·mL^−1^). Macrographs were acquired after 7 days of cultivation.

### Confocal microscopy

Wild‐type *N. oceanica* and *M*. *gaditana*, as well as two independent sMTP‐Sul‐CzBKT lines for each species, were imaged using a Leica Stellaris 8 confocal laser scanning microscope (Leica, Wetzlar, Germany). Prior to microscopy, 50 μL of exponentially growing cultures were transferred onto μ‐slides (18 well ibiTreat; ibidi GmbH, Gräfelfing, Germany), which were previously treated with 0.1% (w/v) Poly‐L‐lysine for 1 h. To reduce movement of the cells, microscopic slides containing cell suspension were spun down at 1000 × ***g*** for 5 min. Chlorophyll autofluorescence and mScarlet‐I3 were excited using a 568 nm supercontinuum white light laser. Emission was detected at 677–762 nm for chlorophyll autofluorescence using a HyD S detector and at 578–640 nm for mScarlet‐I3 using a HyD X detector. Imaging was performed using a 63×/1.3 NA glycerol immersion objective. mScarlet‐I3 fluorescence intensities at the single‐cell level were quantified using ImageJ (version 1.54 g).

### Pigment profile analysis

Algal pigments were extracted from *N*. *oceanica* and *M*. *gaditana* WT cells and two independent transformants harboring either the sMTP‐Sul‐EV or sMTP‐Sul‐CzBKT construct. Microalgal cultures were harvested during exponential growth by centrifugation, and pellets were washed once with deionized water. Pigments were extracted using a liquid–liquid extraction. Pellets were resuspended in 500 μL chloroform: methanol (2:1, v/v), after which 500 μL hexane and 500 μL 5 M NaCl were added. Samples were mixed and centrifuged at 500 × ***g*** for 1 min to achieve phase separation. A 200 μL aliquot of the upper organic phase was evaporated under a gentle stream of N_2_ and re‐dissolved in 100 μL 90% methanol: water (v/v) containing 10 ppm 8‐apo‐carotenal as an internal standard. Before analysis, samples were filtered using a 96‐well filtration plate fitted with a 0.2 μm polyvinylidene fluoride (PVDF) membrane (Agilent Technologies, Santa Clara, CA, USA).

Pigment extracts were analyzed by ultrahigh‐performance liquid chromatography using an Ultimate 3000 UHPLC+ Focused system (Dionex Corporation, Sunnyvale, CA, USA) coupled to an atmospheric pressure chemical ionization quadrupole time‐of‐flight mass spectrometer (Bruker Compact APCI‐QTOF‐MS; Bruker, Billerica, MA, USA) as previously described (Jinkerson et al., 2024). Extracted ion chromatograms of canthaxanthin (m/z 565.4040 [M + H]^+^) and astaxanthin (m/z 597.3989 [M + H]^+^) were detected within ±0.01 m/z and confirmed using authentic standards. Adonirubin (m/z 581.3989 [M + H]^+^) was identified based on accurate mass and comparison to reference absorbance spectra as previously described (Gee et al., 2024).

## Author Contributions

Conceptualization, JS; Funding acquisition, JS; Investigation, JS, CRW, and JP; Methodology, JS; Visualization, JS; Writing – original draft, JS; Writing – review and editing, JS, and JA‐R. All authors have read and agreed to the published version of the manuscript.

## Conflict of Interest

The authors declare no conflicts of interest.

## Supporting information


**Data S1.** Annotated plasmid construct sequences used in this study.

**Data S2.** Source data for all figures.

**Figure S1.** Representative images of *Nannochloropsis* and *Microchloropsis* species grown on agar selection plates containing increasing concentrations of sulfadiazine. *Nannochloropsis oceanica* CCMP1179, *Nannochloropsis oceanica* CCAP 211/78, *Microchloropsis gaditana* CCAP 849/6, and *Microchloropsis salina* CCAP 849/3 were cultured on f/2 N agar plates supplemented with sulfadiazine at 0, 100, 200, 300, 400, 500, or 1000 μg·mL^−1^ for 4 weeks (*n* = 4).
**Figure S2.** Sensitivity of *Nannochloropsis oceanica* and *Microchloropsis gaditana* to sulfadiazine in liquid culture. *N. oceanica* CCMP1779 and *M. gaditana* were cultured under four sulfadiazine concentrations (0, 100, 500, and 1000 μg·mL^−1^). (a) Representative images were acquired 7 days after culture initiation. Growth inhibition in (b) *N. oceanica* and (c) *M. gaditana* in response to sulfadiazine exposure was assessed by optical density measurements at 550 nm (*n* = 3). Data are presented as mean ± SD. Different letters indicate significant differences between time and concentration groups (two‐way ANOVA, Tukey's HSD, *P* ≤ 0.05).
**Figure S3.** Schematic overview of expression constructs used in this study. Sul was targeted to the cytoplasm (Cyto), mitochondria using either a short (sMTP) or long (lMTP) mitochondrial targeting peptide, and to the chloroplast using a chloroplast targeting peptide (cTP). A bidirectional promoter (pRibi) regulated the expression of *β*‐carotene ketolase (*CzBKT*) from *Chromochloris zofingiensis*, fused to the red fluorescence protein *mScarlet‐I3*. The resistance genes contained a downstream Cauliflower mosaic virus 35S terminator (T_35S_), while expression in the opposite direction from pRibi, driving *mScarlet‐I3* or *CzBKT*‐*mScarlet‐I3*, was terminated by an *α*‐tubulin terminator (T_α‐tub_). For comparison, constructs conferring resistance to hygromycin B via aminoglycoside phosphotransferase (*aphVII*) or to Zeocin via the bleomycin resistance gene (*Sh ble*) were used.
**Figure S4.** Colony formation in *Nannochloropsis oceanica* CCMP1779 5 weeks after transformation with empty vector (EV) and CzBKT constructs. (a) Representative control plates on agar supplemented with sulfadiazine (500 μg·mL^−1^), zeocin (2 μg·mL^−1^), or hygromycin B (300 μg·mL^−1^). (b) Representative sulfadiazine selection plates showing transformation efficiency of Sul‐based constructs targeted to the cytoplasm (Cyto), chloroplast (cTP), or mitochondria using either a short or long mitochondrial targeting peptide (sMTP and lMTP, respectively). Colonies were observed only for the sMTP‐Sul‐EV and sMTP‐Sul‐CzBKT constructs, while few or none were detected on the remaining plates. (c) Representative zeocin‐selection plates of *N. oceanica* transformed with constructs carrying the *Sh ble* resistance gene. (d) Representative hygromycin B selection plates of *N. oceanica* transformed with constructs carrying the *aphVII* resistance gene. Transformations are shown for four to six biological replicates (*n* = 4–6). Selective agent‐specific controls (Sul‐C, Zeo‐C, and Hyg‐C) were included with 11–12 replicates per control (*n* = 11–12).
**Figure S5.** Colony formation in *Microchloropsis gaditana* CCAP 849/6 5 weeks after transformation with empty vector (EV) and CzBKT constructs. (a) Representative control plates on agar supplemented with sulfadiazine (500 μg·mL^−1^), zeocin (2 μg·mL^−1^), or hygromycin B (300 μg·mL^−1^). (b) Representative sulfadiazine selection plates showing transformation efficiency of Sul‐based constructs targeted to the cytoplasm (Cyto), chloroplast (cTP), or mitochondria using either a short or long mitochondrial targeting peptide (sMTP and lMTP, respectively). Colonies were observed only for the sMTP‐Sul‐EV and sMTP‐Sul‐CzBKT constructs, while few or none were detected on the remaining plates. (c) Representative zeocin‐selection plates of *M. gaditana* transformed with constructs carrying the *Sh ble* resistance gene. (d) Representative hygromycin B selection plates of *M. gaditana* transformed with constructs carrying the *aphVII* resistance gene. Transformations were performed with five to six biological replicates (*n* = 5–6), and selective agent‐specific controls (Sul‐C, Zeo‐C, Hyg‐C) were included (*n* = 12).
**Figure S6.** *CzBKT* expression in sulfadiazine‐resistant *Microchloropsis gaditana* leads to accumulation of ketocarotenoids. *M. gaditana* harboring the sMTP‐Sul‐CzBKT construct exhibited a pronounced brown phenotype compared with the wild‐type (WT) strain and empty vector control (sMTP‐Sul‐EV). (a) Representative colonies grown on agar plates containing 500 μg·mL^−1^ sulfadiazine, showing strains harboring either the sMTP‐Sul‐EV or sMTP‐Sul‐CzBKT construct. (b) Liquid cultures in f/2 N medium grown in triplicate, including wild‐type controls and transformant lines derived from plate selection. sMTP‐Sul‐CzBKT lines consistently showed a brown phenotype relative to WT and EV controls. Chloroplast‐targeted CzBKT expression was evaluated by single‐cell quantification of mScarlet‐I3 fluorescence intensity in (c, d) *M. gaditana* wild‐type (WT) and sMTP‐Sul‐CzBKT‐expressing lines. Confocal images show chlorophyll autofluorescence (Ex: 568 nm, Em: 676–762 nm) and mScarlet‐I3 (Ex: 568 nm, Em: 578‐640 nm), along with merged channels. The white scale bars represent 10 μm. Fluorescence intensity was analyzed by one‐way ANOVA followed by Tukey's HSD test. Different letters indicate significant differences (*P* ≤ 0.05). Data are shown as mean ± SD with individual data points. (e) *M*. *gaditana* expressing sMTP‐Sul‐CzBKT lines display new carotenoid peaks detected by UV absorbance at 445 nm and confirmed by mass spectrometry, compared with the WT. Peaks corresponding to canthaxanthin (Can., green), adonirubin (Ado., yellow), and astaxanthin (Ast., purple) are highlighted in the chromatogram (*n* = 3). (f) Relative quantification from extracted ion chromatogram (EICs; ±0.01 m/z window) peak area ratios to the internal standard (10 ppm 8‐apo‐carotenal; m/z 417.3152 [M + H]^+^) of canthaxanthin (m/z 565.4040 [M + H]^+^), adonirubin (m/z 581.3989 [M + H]^+^) and astaxanthin (m/z 597.3989 [M + H]^+^) in two independent sMTP‐Sul‐CzBKT lines, two independent sMTP‐Sul‐EV lines and the WT in *M*. *gaditana*. Data are presented as mean ± SD (*n* = 3). Different letters indicate statistically significant differences among groups for each metabolite, as determined by one‐way ANOVA followed by Tukey's HSD test (*P* ≤ 0.05). n.d., not detected.
**Table S1.** Reported antibiotic concentrations used in the literature for genetic transformation in *Nannochloropsis* spp. and *Microchloropsis* spp.
**Table S2.** BlastP identification of DHPS homologs in eustigmatophyte microalgae using mitochondrial *Arabidopsis thaliana* DHPS (At4g30000), followed by subcellular localization prediction with DeepLoc v2.1.
**Table S3.** Primers used in this study.
**Table S4.** Overview of sequences used for construction of plasmids.
**Method S1.** Sulfadiazine dose–response in liquid cultures of *Nannochloropsis oceanica* CCMP1779 and *Microchloropsis gaditana*.

## Data Availability

All data supporting the findings of this study are included in this article and its Supporting Information.
