# Supplementary material for: Sulfadiazine selection enables high‐efficiency transformation in Nannochloropsis and Microchloropsis
Source: Plant J. 2026 Jul 31;127(3):e71063. doi: 10.1111/tpj.71063 (PMC13427358; doi:10.1111/tpj.71063)
Supplement: Supplementary file 3 — Figure S1. Representative images of Nannochloropsis and Microchloropsis species grown on agar selection plates containing increasing concentrations of sulfadiazine. Nannochloropsis oceanica CCMP1179, Nannochloropsis oceanica CCAP 211/78, Microchloropsis gaditana CCAP 849/6, and Microchloropsis salina CCAP 849/3 were cultured on f/2 N agar plates supplemented with sulfadiazine at 0, 100, 200, 300, 400, 500, or 1000 μg·mL−1 for 4 weeks (n = 4). Figure S2. Sensitivity of Nannochloropsis oceanica and Microchloropsis gaditana to sulfadiazine in liquid culture. N. oceanica CCMP1779 and M. gaditana were cultured under four sulfadiazine concentrations (0, 100, 500, and 1000 μg·mL−1). (a) Representative images were acquired 7 days after culture initiation. Growth inhibition in (b) N. oceanica and (c) M. gaditana in response to sulfadiazine exposure was assessed by optical density measurements at 550 nm (n = 3). Data are presented as mean ± SD. Different letters indicate significant differences between time and concentration groups (two‐way ANOVA, Tukey's HSD, P ≤ 0.05). Figure S3. Schematic overview of expression constructs used in this study. Sul was targeted to the cytoplasm (Cyto), mitochondria using either a short (sMTP) or long (lMTP) mitochondrial targeting peptide, and to the chloroplast using a chloroplast targeting peptide (cTP). A bidirectional promoter (pRibi) regulated the expression of β‐carotene ketolase (CzBKT) from Chromochloris zofingiensis, fused to the red fluorescence protein mScarlet‐I3. The resistance genes contained a downstream Cauliflower mosaic virus 35S terminator (T35S), while expression in the opposite direction from pRibi, driving mScarlet‐I3 or CzBKT‐mScarlet‐I3, was terminated by an α‐tubulin terminator (Tα‐tub). For comparison, constructs conferring resistance to hygromycin B via aminoglycoside phosphotransferase (aphVII) or to Zeocin via the bleomycin resistance gene (Sh ble) were used. Figure S4. Colony formation in Nannochloropsis oceanica [file TPJ-127-0-s001.docx]

# **Supporting Information**

**Method S1. Sulfadiazine dose-response in liquid cultures of *Nannochloropsis* *oceanica* CCMP1779 and *Microchloropsis gaditana***

Twenty milliliters of wild-type *N. oceanica* and *M. gaditana* were inoculated at an initial OD_550_ ≈ 0.5 and grown in f/2N medium supplemented with 0, 100, 500, or 1000 µg·mL^-1^ sulfadiazine in triplicate. After seven days, cell growth was assessed by measuring optical density at 550 nm, and macrographs of the cultures acquired.


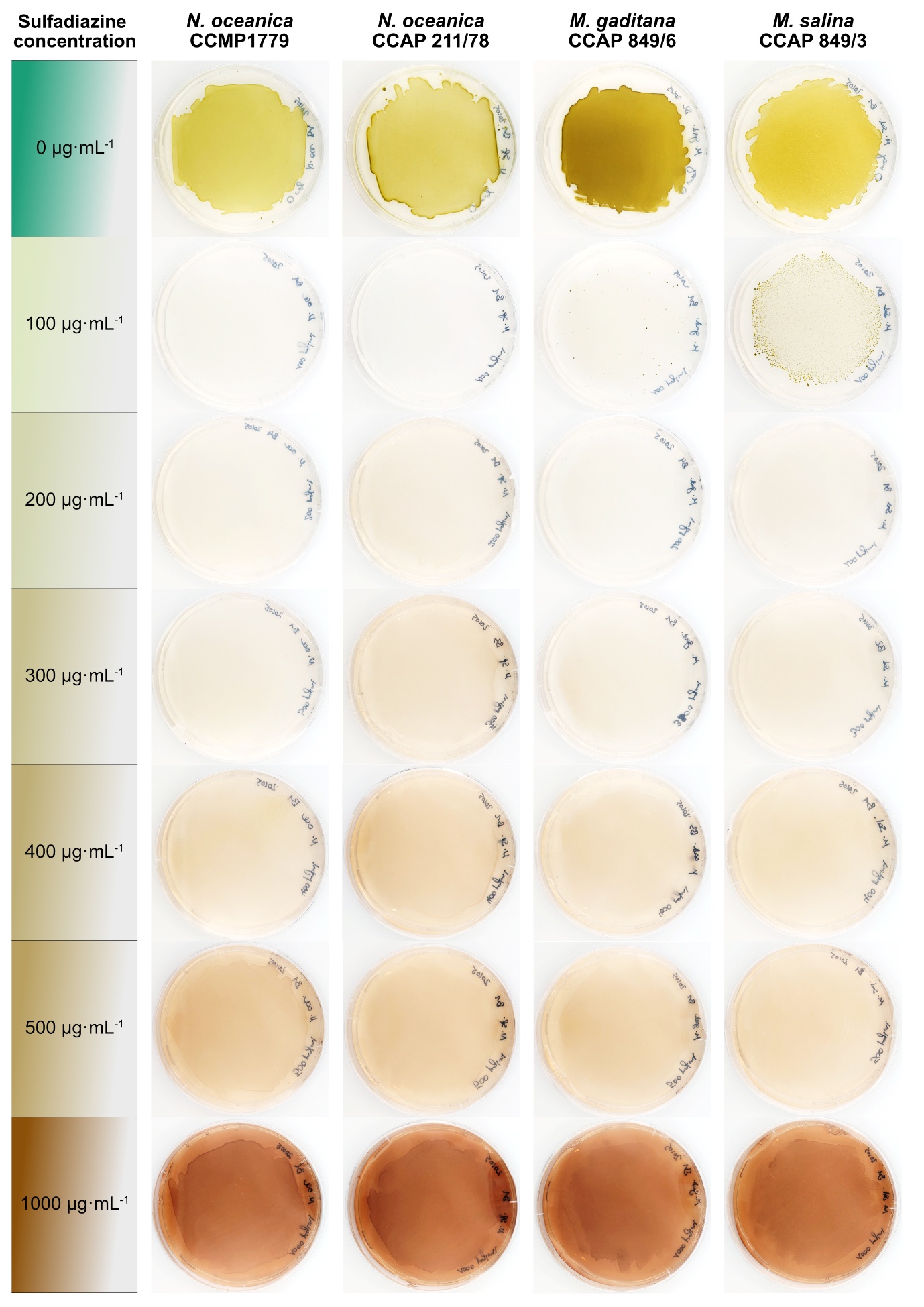


**Figure S1. Representative images of *Nannochloropsis* and *Microchloropsis* species grown on agar selection plates containing increasing concentrations of sulfadiazine.** *Nannochloropsis oceanica* CCMP1179, *Nannochloropsis oceanica* CCAP 211/78, *Microchloropsis gaditana* CCAP 849/6, and *Microchloropsis salina* CCAP 849/3 were cultured on f/2N agar plates supplemented with sulfadiazine at 0, 100, 200, 300, 400, 500, or 1000 μg·mL^-1^ for four weeks (*n* = 4).


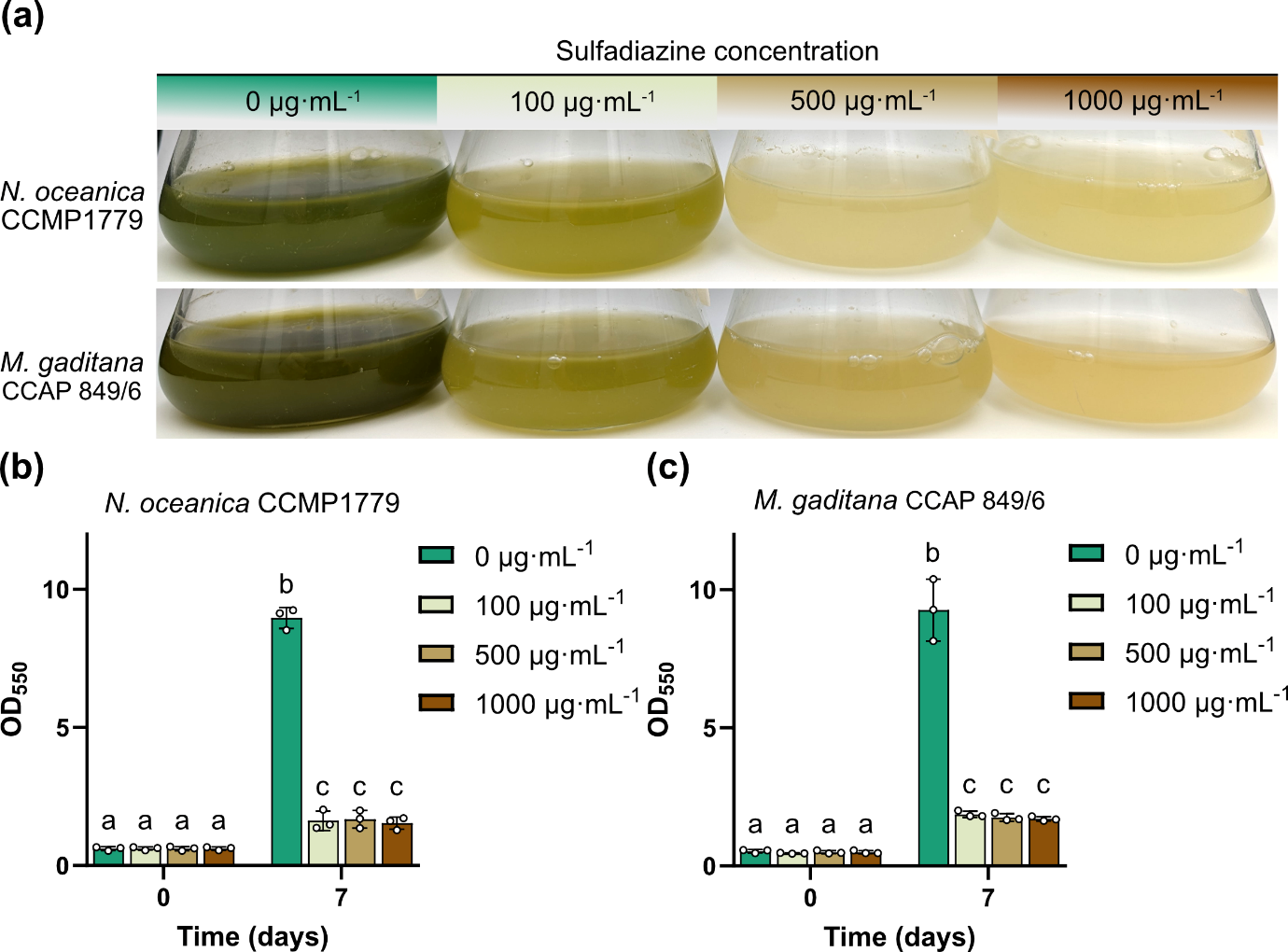


**Figure S2. Sensitivity of *Nannochloropsis oceanica* and *Microchloropsis gaditana* to sulfadiazine in liquid culture.** *N. oceanica* CCMP1779 and *M. gaditana* were cultured under four sulfadiazine concentrations (0, 100, 500, and 1000 µg·mL^-1^). **(a)** Representative images were acquired seven days after culture initiation. Growth inhibition in **(b)** *N. oceanica* and **(c)** *M. gaditana* in response to sulfadiazine exposure was assessed by optical density measurements at 550 nm (*n* = 3). Data are presented as mean ± SD. Different letters indicate significant differences between time and concentration groups (two-way ANOVA, Tukey’s HSD, *p* ≤ 0.05).


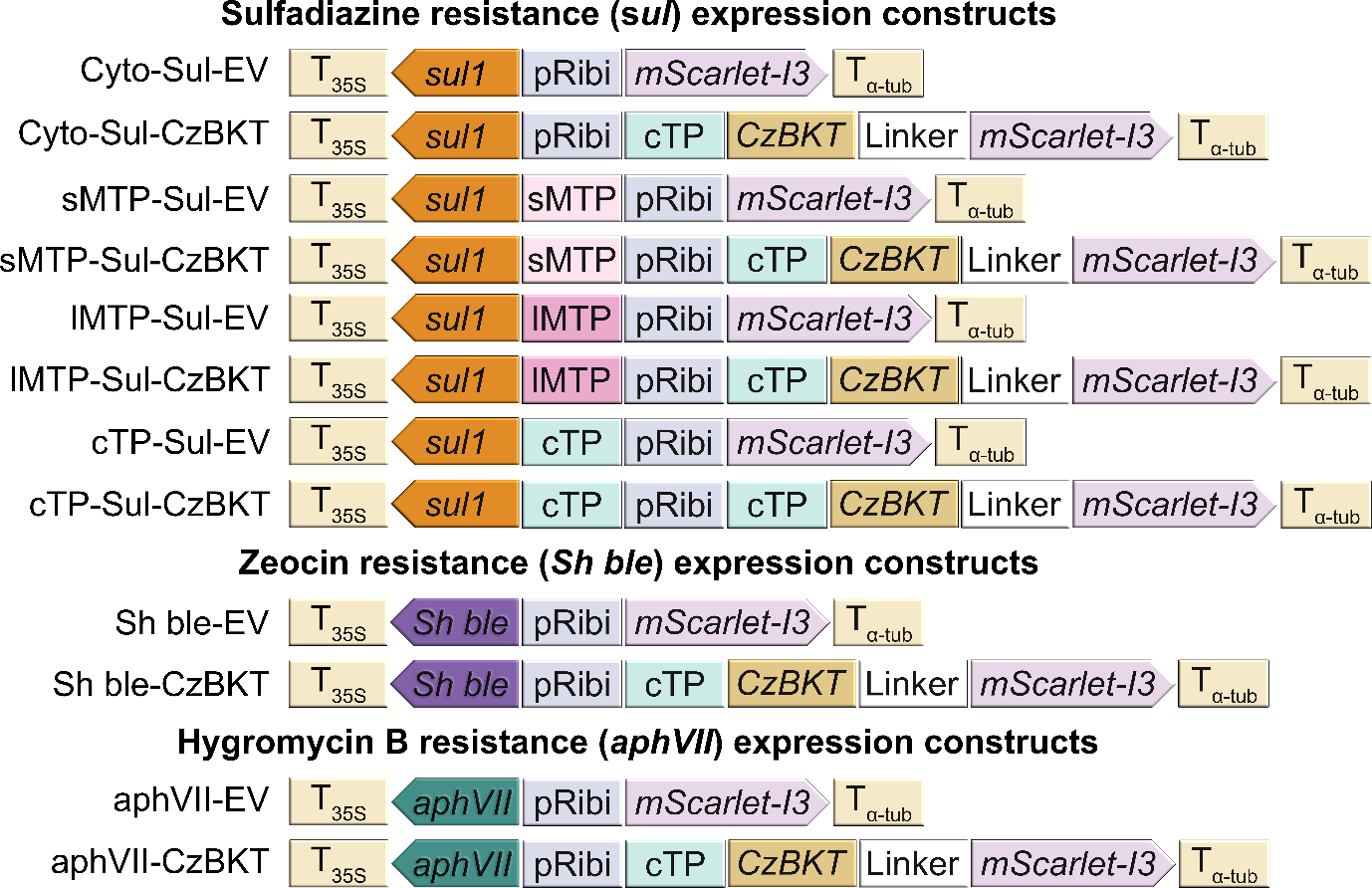


**Figure S3. Schematic overview of expression constructs used in this study.** Sul was targeted to the cytoplasm (Cyto), mitochondria using either a short (sMTP) or long (lMTP) mitochondrial targeting peptide, and to the chloroplast using a chloroplast targeting peptide (cTP). A bidirectional promoter (pRibi) regulated the expression of *β*-carotene ketolase (*CzBKT*) from *Chromochloris zofingiensis*, fused to the red fluorescence protein *mScarlet-I3*. The resistance genes contained a downstream Cauliflower mosaic virus 35S terminator (T_35S_), while expression in the opposite direction from pRibi, driving *mScarlet-I3* or *CzBKT*-*mScarlet-I3*, was terminated by an *α*-tubulin terminator (T_α-tub_). For comparison, constructs conferring resistance to hygromycin B via aminoglycoside phosphotransferase (*aphVII*) or to Zeocin via the bleomycin resistance gene (*Sh ble*) were used.


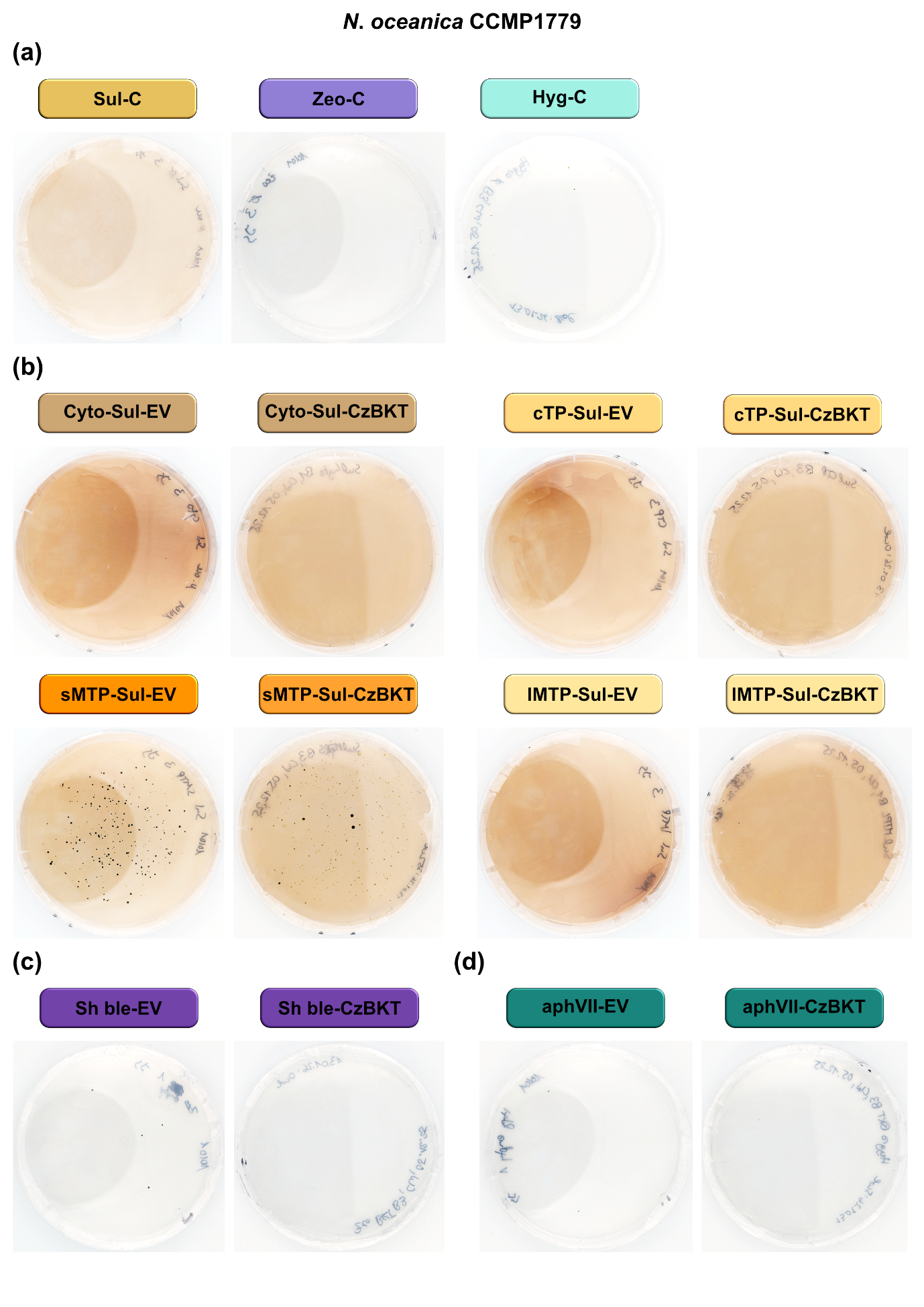


**Figure S4. Colony formation in *Nannochloropsis oceanica* CCMP1779 five weeks after transformation with empty vector (EV) and CzBKT constructs. (a)** Representative control plates on agar supplemented with sulfadiazine (500 µg·mL^-1^), zeocin (2 µg·mL^-1^), or hygromycin B (300 µg·mL^-1^). **(b)** Representative sulfadiazine-selection plates showing transformation efficiency of Sul-based constructs targeted to the cytoplasm (Cyto), chloroplast (cTP), or mitochondria using either a short or long mitochondrial targeting peptide (sMTP and lMTP, respectively). Colonies were observed only for the sMTP-Sul-EV and sMTP-Sul-CzBKT constructs, while few or none were detected on the remaining plates. **(c)** Representative zeocin-selection plates of *N. oceanica* transformed with constructs carrying the *Sh ble* resistance gene. **(d)** Representative hygromycin B-selection plates of *N. oceanica* transformed with constructs carrying the *aphVII* resistance gene. Transformations are shown for four to six biological replicates (*n* = 4-6). Selective agent-specific controls (Sul-C, Zeo-C, and Hyg-C) were included with 11–12 replicates per control (*n* = 11–12).


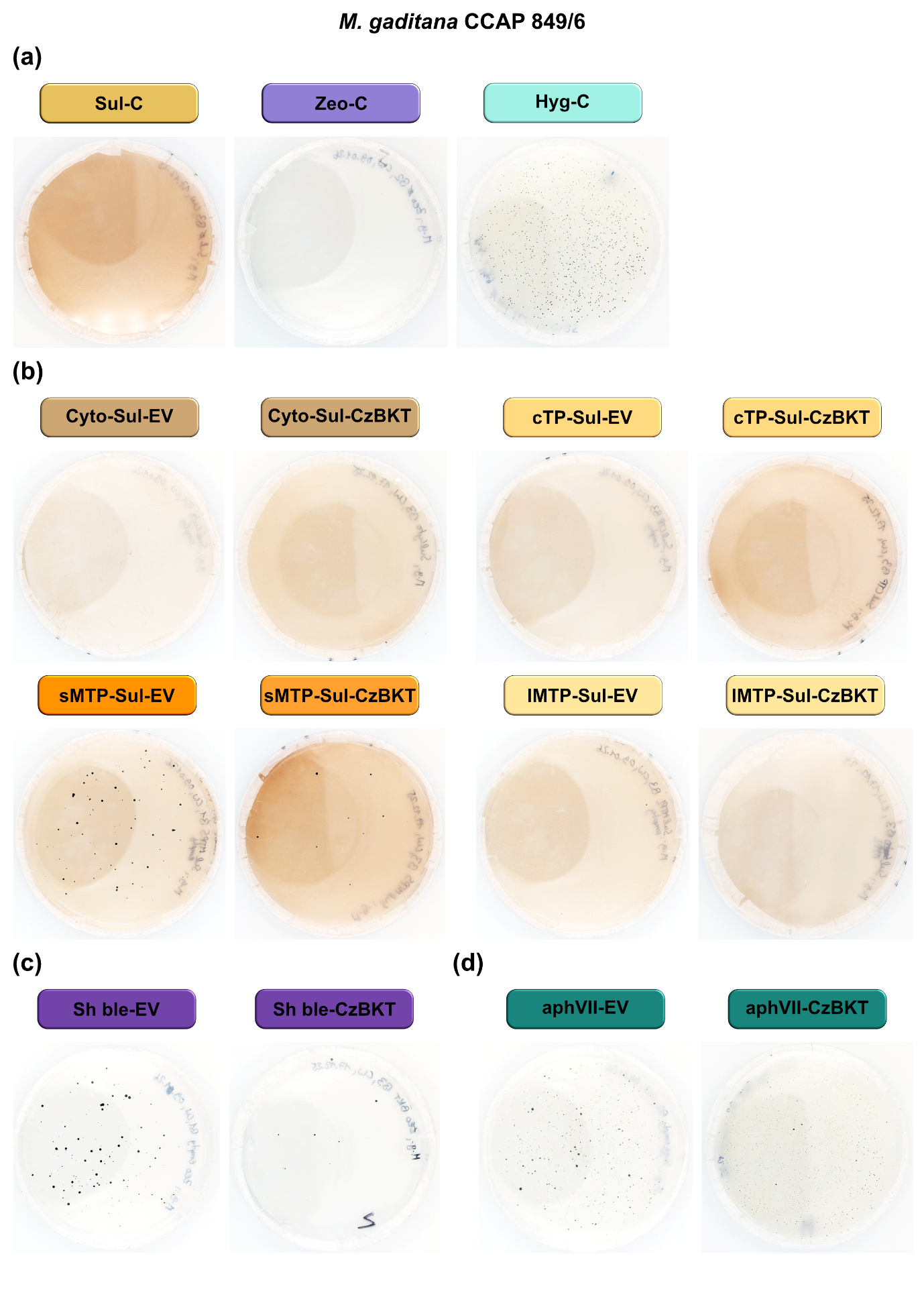


**Figure S5. Colony formation in *Microchloropsis gaditana* CCAP 849/6 five weeks after transformation with empty vector (EV) and CzBKT constructs. (a)** Representative control plates on agar supplemented with sulfadiazine (500 µg·mL^-1^), zeocin (2 µg·mL^-1^), or hygromycin B (300 µg·mL^-1^). **(b)** Representative sulfadiazine-selection plates showing transformation efficiency of Sul-based constructs targeted to the cytoplasm (Cyto), chloroplast (cTP), or mitochondria using either a short or long mitochondrial targeting peptide (sMTP and lMTP, respectively). Colonies were observed only for the sMTP-Sul-EV and sMTP-Sul-CzBKT constructs, while few or none were detected on the remaining plates. **(c)** Representative zeocin-selection plates of *M. gaditana* transformed with constructs carrying the *Sh ble* resistance gene. **(d)** Representative hygromycin B-selection plates of *M. gaditana* transformed with constructs carrying the *aphVII* resistance gene. Transformations were performed with five to six biological replicates (*n* = 5–6), and selective agent-specific controls (Sul-C, Zeo-C, Hyg-C) were included (*n* = 12).


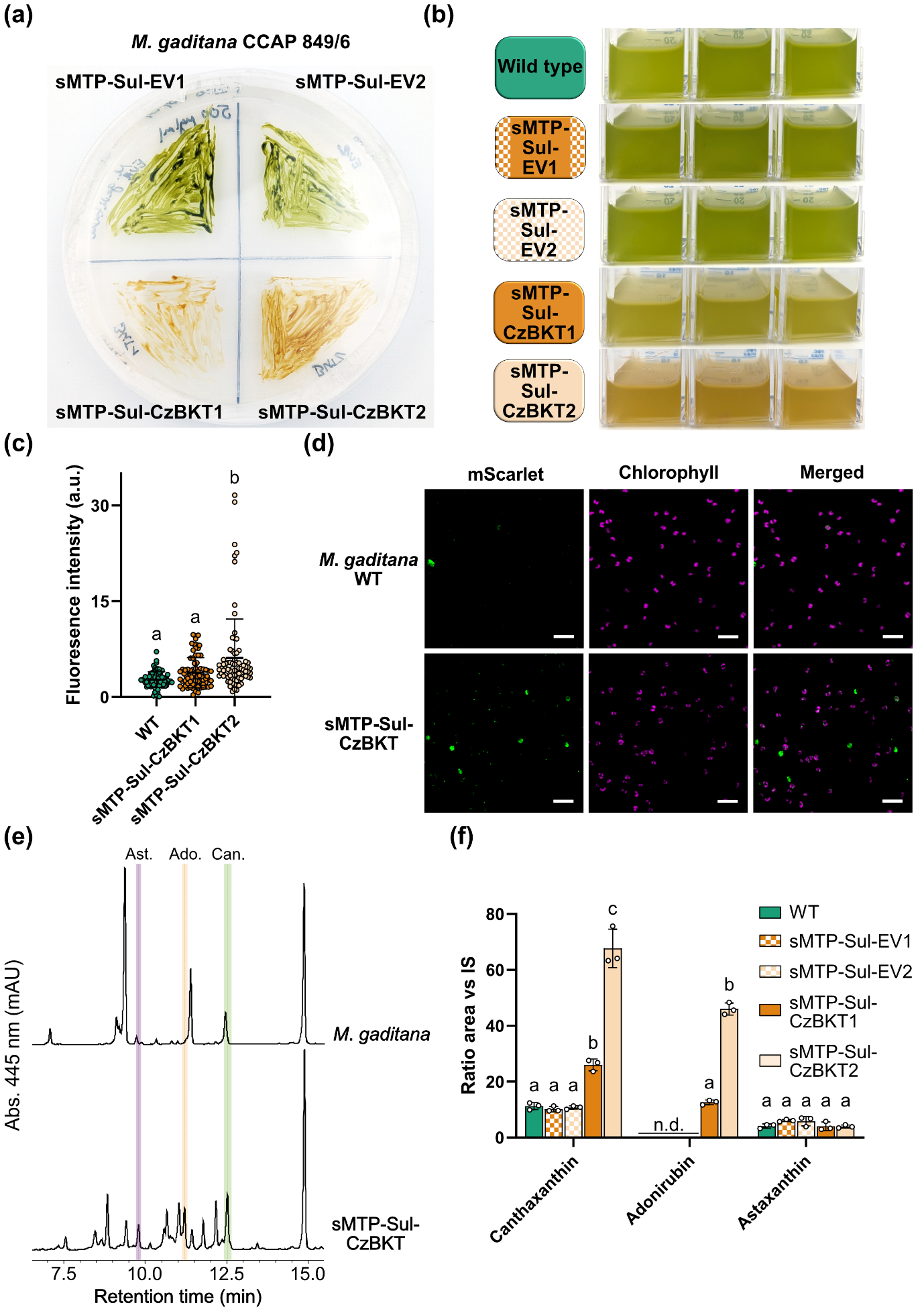


**Figure S6. *CzBKT* expression in sulfadiazine resistant *Microchloropsis gaditana* leads to accumulation of ketocarotenoids.** *M. gaditana* harboring the sMTP-Sul-CzBKT construct exhibited a pronounced brown phenotype compared to the wild-type (WT) strain and empty vector control (sMTP-Sul-EV). **(a)** Representative colonies grown on agar plates containing 500 µg·mL^-1^ sulfadiazine, showing strains harboring either the sMTP-Sul-EV or sMTP-Sul-CzBKT construct. **(b)** Liquid cultures in f/2N medium grown in triplicate, including wild-type controls and transformant lines derived from plate selection. sMTP-Sul-CzBKT lines consistently showed a brown phenotype relative to WT and EV controls. Chloroplast-targeted CzBKT expression was evaluated by single-cell quantification of mScarlet-I3 fluorescence intensity in **(c-d)** *M. gaditana* wild type (WT) and sMTP-Sul-CzBKT-expressing lines. Confocal images show chlorophyll autofluorescence (Ex: 568 nm, Em: 676-762 nm) and mScarlet-I3 (Ex: 568 nm, Em: 578-640nm), along with merged channels. The white scale bars represent 10 µm. Fluorescence intensity was analyzed by one-way ANOVA followed by Tukey’s HSD test. Different letters indicate significant differences (*p* ≤ 0.05). Data are shown as mean ± SD with individual data points. **(e)** *M.* *gaditana* expressing sMTP-Sul-CzBKT lines display new carotenoid peaks detected by UV absorbance at 445 nm and confirmed by mass spectrometry, compared with the WT. Peaks corresponding to canthaxanthin (Can., green), adonirubin (Ado., yellow), and astaxanthin (Ast., purple) are highlighted in the chromatogram (*n* = 3). **(f)** Relative quantification from extracted ion chromatogram (EICs; ±0.01 m/z window) peak area ratios to the internal standard (10 ppm 8-apo-carotenal; m/z 417.3152 [M+H]^+^) of canthaxanthin (m/z 565.4040 [M+H]^+^), adonirubin (m/z 581.3989 [M+H]^+^) and astaxanthin (m/z 597.3989 [M+H]^+^) in two independent sMTP-Sul-CzBKT lines, two independent sMTP-Sul-EV lines and the WT in *M*. *gaditana*. Data are presented as mean ± SD (*n* = 3). Different letters indicate statistically significant differences among groups for each metabolite, as determined by one-way ANOVA followed by Tukey’s HSD test (*p* ≤ 0.05). n.d., not detected.

**Table S1. Reported antibiotic concentrations used in the literature for genetic transformation in *Nannochloropsis* spp. and *Microchloropsis* spp..**

| **Selective agent** | **Species** | **Strain** | **Concentration (µg**·**mL^-1^)** | **References** |
| --- | --- | --- | --- | --- |
| **Hygromycin B** | *N. granulata* | CCMP529 | >200 | (Vieler *et al.*, 2012) |
|  | *N. oceanica* | CCMP531 | 50 | (Vieler *et al.*, 2012) |
|  | *N. oceanica* | CCMP1779 | 25-500 | (Poliner *et al.*, 2018; Poliner *et al.*, 2020; Poveda-Huertes *et al.*, 2023; Vieler *et al.*, 2012) |
|  | *N. oceanica* | IMET1 | 300 | (Liu *et al.*, 2024; Wang *et al.*, 2016) |
|  | *N. oceanica* | NIES-2145 | 100 | (Moroi *et al.*, 2025) |
|  | *N.* sp. | W2J3B | 300 | (Kilian *et al.*, 2011) |
|  | *M. gaditana* | CCMP536 | >100 | (Vieler *et al.*, 2012) |
|  | *M. gaditana* | CCMP1775 | >100 | (Vieler *et al.*, 2012) |
|  | *M. gaditana* | CCMP1894 | 500 | (Ajjawi *et al.*, 2017; Verruto *et al.*, 2018) |
|  | *M. salina* | CCMP369 | >200 | (Vieler *et al.*, 2012) |
|  | *M. salina* | CCM537 | Resistant | (Poliner *et al.*, 2019) |
| **Zeocin** | *N. granulata* | CCMP529 | 5 | (Vieler *et al.*, 2012) |
|  | *N. oceanica* | CCMP531 | 5 | (Vieler *et al.*, 2012) |
|  | *N. oceanica* | CCMP1779 | 5 | (Poliner *et al.*, 2020; Vieler *et al.*, 2012) |
|  | *N. oceanica* | IMET1 | 2-10 | (Liu *et al.*, 2022; Liu *et al.*, 2023; Liu *et al.*, 2024; Naduthodi *et al.*, 2019; Naduthodi *et al.*, 2021; Südfeld *et al.*, 2021; Südfeld *et al.*, 2022a; Südfeld *et al.*, 2022b; Südfeld *et al.*, 2023; Zhu *et al.*, 2025) |
|  | *N. oceanica* | NIES-2145 | 2 | (Kilian *et al.*, 2011; Kurita *et al.*, 2020; Moroi *et al.*, 2025) |
|  | *N.* sp. | W2J3B | 2 | (Kilian *et al.*, 2011) |
|  | *M. gaditana* | CCAP 849/5 | 3.5 | (Perin *et al.*, 2015) |
|  | *M. gaditana* | CCMP526 | 30 | (Vogler *et al.*, 2021) |
|  | *M. gaditana* | CCMP536 | 5 | (Vieler *et al.*, 2012) |
|  | *M. gaditana* | CCMP1775 | 5 | (Vieler *et al.*, 2012) |
|  | *M. gaditana* | CCMP1894 | 10 | (Verruto *et al.*, 2018) |
|  | *M. salina* | CCMP369 | 5 | (Vieler *et al.*, 2012) |
|  | *M. salina* | CCMP1776 | 2.5 | (Jeong *et al.*, 2020) |
| **Blasticidin S** | *N. oceanica* | CCMP1779 | 25 | (Poliner *et al.*, 2020) |
|  | *N. oceanica* | IMET1 | 100 | (Michelberger *et al.*, 2025; Naduthodi *et al.*, 2021; Südfeld *et al.*, 2022a; Südfeld *et al.*, 2022b) |
|  | *N.* sp. | W2J3B | 50 | (Kilian *et al.*, 2011) |
|  | *M. gaditana* | CCMP1894 | 100 | (Verruto *et al.*, 2018) |
| **G418** | *N. oceanica* | CCMP1779 | 250 | (Du *et al.*, 2023; Poliner *et al.*, 2020) |
|  | *M. salina* | CCMP1776 | 100 | (Jeong *et al.*, 2020) |
| **Nourseothricin** | *N. oceanica* | CCMP1779 | 200 | (Poliner *et al.*, 2020; Poliner *et al.*, 2022) |
| **Norflurazon** | *N. oceanica* | IMET1 | 4.5 | (Liu *et al.*, 2022) |

**Table S2. BlastP identification of DHPS homologs in eustigmatophyte microalgae using mitochondrial *Arabidopsis thaliana* DHPS (At4g30000), followed by subcellular localization prediction with DeepLoc v2.1.**

| **Species** | **Strain** | **Identifier** | **Predicted localization** | **Probability** |
| --- | --- | --- | --- | --- |
| *Arabidopsis thaliana* | Col-0 | At4g30000 | mitochondrial | 0.79 |
| *Arabidopsis thaliana* | Col-0 | At1g69190 | Cytosolic | 0.78 |
| *Nannochloropsis oceanica* | CCMP1779 v2.0 | 608533 | mitochondrial | 0.88 |
| *Nannochloropsis oceanica* | IMET1 | NO23G01830 | mitochondrial | 0.86 |
| *Nannochloropsis oceanica* | C018 v1.0 | 300962 | mitochondrial | 0.82 |
| *Nannochloropsis oceanica* | CCAP849/10 | 9655 | mitochondrial | 0.85 |
| *Microchloropsis gaditana* | CCMP1894 | 2621 | cytosolic/mitochondrial | 0.52/0.86 |
| *Microchloropsis gaditana* | B-31 | EWM27776.1 | cytosolic | 0.78 |
| *Microchloropsis salina* | CCMP1776 | 1088 | mitochondrial | 0.89 |
| *Monodopsis* | C73 | 7703 | mitochondrial | 0.60 |
| *Monodopsis* | C141 | 3815 | mitochondrial | 0.81 |
| *Vischeria* | C74 | 6960 | cytosolic | 0.52 |

**Table S3.** **Primers used in this study.**

| **Primer** | **Description** | **Sequence** |
| --- | --- | --- |
| GOI_seq_F | Sequencing primer gene of interest | TGTTGCAGTGGCGTCATCCT |
| GOI_seq_R | Sequencing primer gene of interest | TCATGGAGCCCTCCATGTGG |
| Resistance_seq_F | Sequencing primer resistance gene | TGAGCGAAACCCTATAAGAAC |
| Resistance_seq_R | Sequencing primer resistance gene | AAATCGGTGGTCAACAACAGA |
| pABNE_linearize_F | Linearization of pABNE expression fragment for transformation | CATATGCAGGTCACTGGATTTTG |
| pABNE_linearize _R | Linearization pABNE expression fragment for transformation | GATATCAAGGGAGGGTAGTGG |
| cTP-*CzBKT*_clone_F | Cloning cTP-*CzBKT* (AY772713) into *HindIII* digested pABNE-Zeov1, pABNE-Sulv1, pABNE-Hygv1 | ccacagagcgcaggcctatATGAAGACCGCCGCTCTC |
| cTP-*CzBKT*_clone_R | Cloning c*TP*-*CzBKT* (AY772713) into *HindIII* digested pABNE-Zeov1, pABNE-Sulv1, pABNE-Hygv1 | ccagcactaccagcactaccGTTCACCAACTGACTCTTCTTCG |
| *aphVII*_clone_F | Cloning *aphVII* into *SpeI* digested pABNE-Zeov1 | tccggtcggcatctactactCTATTCCTTTGCCCTCGGA |
| *aphVII*_clone_R | Cloning *aphVII* into SpeI digested pABNE-Zeov1 | cagatacacacgcaaccaTGAAAAAGCCTGAACTCACCG |
| Cyto-*sul1*_clone_F | Cloning Cyto-*sul1* into *SpeI* digested pABNE-Zeov1 | tccggtcggcatctactactCTAGGCATGATCTAACCCT |
| Cyto-*sul1*_clone_R | Cloning Cyto-*sul1* into *SpeI* digested pABNE-Zeov1 | cagatacacacgcaaccaTGGTGACGGTGTTCGGCATT |
| cTP_clone_F | Amplification of cTP derived from *NoVCP1* for chloroplast localization of Sul | cgtcaccatGCGCGCAACACCGC |
| cTP_clone_R | Amplification of cTP derived from *NoVCP1* for chloroplast localization of Sul | cagatacacacgcaaccaATGAAGACCGCCGCTCTC |
| cTP-*sul1*_clone_F | Amplification sul for cloning cTP_*sul1* into *SpeI* digested pABNE-Zeov1 | tccggtcggcatctactaCTAGGCATGATCTAACCCTCGG |
| cTP-*sul1*_clone_R | Amplification sul for cloning cTP-*sul1* into *SpeI* digested pABNE-Zeov1 | gttgcgcgcATGGTGACGGTGTTCGGCAT |
| sMTP-clone_F | Amplification of short mitochondrial targeting peptide (sMTP) derived from *NoOXA1* for mitochondrial localization of Sul | cgtcaccatGGGTACTGAGGAGGAGACGC |
| lMTP-clone_F | Amplification of long mitochondrial targeting peptide (lMTP) derived from *NoOXA1* for mitochondrial localization of Sul | cgtcaccatGATGGAGTCGTAGATCCAGGC |
| sMTP/lMTP-clone_R | Amplification of short and long mitochondrial targeting peptide (sMTP/lMTP) derived from *NoOXA1* for mitochondrial localization of Sul | cagatacacacgcaaccaATGATGAGGCTGGGGGGC |
| sMTP/lMTP-*sul1*_clone_F | Amplification *sul1* for cloning sMTP-*sul1* and lMTP-*sul1* into *SpeI* digested pABNE-Zeov1 | tccggtcggcatctactaCTAGGCATGATCTAACCCTCGG |
| lMTP-*sul1*_clone_R | Amplification *sul1* for cloning  lMTP-*sul1* into *SpeI* digested pABNE-Zeov1 | cagatacacacgcaaccaATGGTGACGGTGTTCGGCAT |
| sMTP-*sul1*_clone_R | Amplification *sul1* for cloning  sMTP-*sul1* into *SpeI* digested pABNE-Zeov1 | tcagtacccATGGTGACGGTGTTCGGCAT |

**Table S4. Overview of sequences used for construction of plasmids.**

| **Gene** | **Database** | **Identifier** | | **Description** | | **Sequence** | | **Reference** | |
| --- | --- | --- | --- | --- | --- | --- | --- | --- | --- |
| *sMTP* | PhycoCosm | 589186 | | Short mitochondrial targeting peptide from *NoOXA1* (oxidase assembly protein 1) | | >sMTP (CCMP1779 v2.0 ID 589186)  ATGATGAGGCTGGGGGGCCAAGGCACGAAGATCCTTTCAAGACGGCGGCCACCACCATCACCAAGCGCAGCAGAAGCAGTACATTCAAGACAAGGAGCAAAAGCAGTGCGGCAACATTTATCAGCATGCATGGCAACATTTTCCCCTTCACACCGCAGAGCCCATCGCCTCGCCCCAACCTTTGCCCTGTCGTCGTACCGAGCATTTAGCGTCTCCTCCTCAGTACCC | | This study | |
| *lMTP* | PhycoCosm | 589186 | | Long mitochondrial targeting peptide from *NoOXA1* (oxidase assembly protein 1) | | >lMTP (CCMP1779 v2.0 ID 589186)  ATGATGAGGCTGGGGGGCCAAGGCACGAAGATCCTTTCAAGACGGCGGCCACCACCATCACCAAGCGCAGCAGAAGCAGTACATTCAAGACAAGGAGCAAAAGCAGTGCGGCAACATTTATCAGCATGCATGGCAACATTTTCCCCTTCACACCGCAGAGCCCATCGCCTCGCCCCAACCTTTGCCCTGTCGTCGTACCGAGCATTTAGCGTCTCCTCCTCAGTACCCACCACCGATCTCTCAACTTCACCACTATCGCATTCGTCGTTGGAAGATTCTACCACCTGGCACGCCTGGATCTACGACTCCATC | | (Moog *et al.*, 2015) | |
| *cTP* | PhycoCosm | 603160 | | Chloroplast targeting peptide derived from *NoVCP1* (violaxanthin/chlorophyll a-binding protein 1) | | >c*TP* (CCMP1779 v2.0 ID 603160)  ATGAAGACCGCCGCTCTCCTCACTGTCTCCACCCTCATGGGCGCCCAGGCCTTTATGGCCCCCGCCCCCAAGTTCTCCCGCACCCGCGGTGTTGCGCGC | | (Moog *et al.*, 2015) | |
| *sul1* | Addgene | pMpGWBs00 | | Sulfadiazine resistant dihydropteroate synthase (Sul) | | >*sul1*  ATGGTGACGGTGTTCGGCATTCTGAATCTCACCGAGGACTCCTTCTTCGATGAGAGCCGGCGGCTAGACCCCGCCGGCGCTGTCACCGCGGCGATCGAAATGCTGCGAGTCGGATCAGACGTCGTGGATGTCGGACCGGCCGCCAGCCATCCGGACGCGAGGCCTGTATCGCCGGCCGATGAGATCAGACGTATTGCGCCGCTCTTAGACGCCCTGTCCGATCAGATGCACCGTGTTTCAATCGACAGCTTCCAACCGGAAACCCAGCGCTATGCGCTCAAGCGCGGCGTGGGCTACCTGAACGATATCCAAGGATTTCCTGACCCTGCGCTCTATCCCGATATTGCTGAGGCGGACTGCAGGCTGGTGGTTATGCACTCAGCGCAGCGGGATGGCATCGCCACCCGCACCGGTCACCTTCGACCCGAAGACGCGCTCGACGAGATTGTGCGGTTCTTCGAGGCGCGGGTTTCCGCCTTGCGACGGAGCGGGGTCGCTGCCGACCGGCTCATCCTCGATCCGGGGATGGGATTTTTCTTGAGCCCCGCACCGGAAACATCGCTGCACGTGCTGTCGAACCTTCAAAAGCTGAAGTCGGCGTTGGGGCTTCCGCTATTGGTCTCGGTGTCGCGGAAATCCTTCTTGGGCGCCACCGTTGGCCTTCCTGTAAAGGATCTGGGTCCAGCGAGCCTTGCGGCGGAACTTCACGCGATCGGCAATGGCGCTGACTACGTCCGCACCCACGCGCCTGGAGATCTGCGAAGCGCAATCACCATCTCGGAAACCCTCGCGAAATTTCGCAGTCGCGACGCCAGAGACCGAGGGTTAGATCATGCCTAG | | (Robinson *et al.*, 2024) | |
| *CzBKT* | NCBI | AY772713 | | *β-carotene ketolase* (*CzBKT*) from *Chromochloris zofingiensis* | | >*CzBKT* (AY772713)  ATGGCGCCAGATGTGACACATGTGCAGCCACGTGTACAGTCCCCGGCTGGCCCCGATGATGAGGATGACGCGTTAAGCTTGTGGAAAGCCCAATATCCTATGCCGGAGGAGAAGGGTACAGTATCCAAACCTCAAGCCGCACTCAAATACAGGCCACCACGCAGTGACTGGAAGGGTGTATCAATTGCATGCACTGTCATCACCCTATGGACAGCTGTCTTTTACCATGGCTGCTGGCAAATCAAACTCACAGGCCCTGATAAGTCAGCCTGGTGGGACGTTGTTGCAACGTTTCTGGCACTGGAGTTCCTCAACACTGGGCTTTTCATCACCACGCATGATGCCATGCATGGGACTATTGCCATCAGGAACCGTCGTTTGAATGACCTACTTGGCAATATAGCCATCAGCCTATATGCCTGGTTTGACTATGACATGCTGCACAAGAAGCACTGGGAGCATCACAACTTCACTGGGTTACCACATAAAGACCCAGACTTCCATCGAGGCGATCCTGCGCTACATAAGTGGTTTGGCAGGTTTATGTGGGAGTATGCAACACCACTCCAGTTTGCCAAGATCTTCGCATACACCTTCTTCCTACAATCCTTACGGGTGCAATACCCCAATTTATGCGTCTTTCTGGCGGCTGCACCCCTGGTCAGTGCGTTCCGATTGTTCTATTTTGGCACCTATTTACCCCACCTCCCCTCCAATGCTCAGGAGACAATGCCCTGGGAGAAATCTCACAGTGCTGATGACCCTCGGCCGCTGTCATTCTTGAAATGTTATCACTTTGATTATCACTGGGAGCATCACAGGTGGCCTTATGCCCCTTGGTGGGAGTTACCCGTGTGTAAGCGCATCACAAAGACACTGGATGCTGCAGTTCCAGGAGTACAGTCAGACGGCACGAAGAAGAGTCAGTTGGTGAACTAA | | (Roth *et al.*, 2017) | |
|  |  |  |  | |  | |  | |  |

**Supporting Information References**

**Ajjawi, I., Verruto, J., Aqui, M., Soriaga, L.B., Coppersmith, J., Kwok, K. et al.** (2017) Lipid production in *Nannochloropsis gaditana* is doubled by decreasing expression of a single transcriptional regulator. *Nature Biotechnology*, 35, 647–652. Available from: https://doi.org/10.1038/nbt.3865

**Du, Z.‐Y., Bhat, W.W., Poliner, E., Johnson, S., Bertucci, C., Farre, E. et al.** (2023) Engineering *Nannochloropsis oceanica* for the production of diterpenoid compounds. *mLife*, 2, 428–437. Available from: https://doi.org/10.1002/mlf2.12097

**Jeong, S.W., HwangBo, K., Lim, J.M., Nam, S.W., Lee, B.S., Jeong, B.‐R. et al.** (2020) Genetic impairment of cellulose biosynthesis increases cell wall fragility and improves lipid extractability from oleaginous alga *Nannochloropsis salina*. *Microorganisms*, 8, 1–14. Available from: https://doi.org/10.3390/microorganisms8081195

**Kilian, O., Benemann, C.S.E., Niyogi, K.K. & Vick, B.** (2011) High‐efficiency homologous recombination in the oil‐producing alga *Nannochloropsis* sp. *Proceedings of the National Academy of Sciences of the United States of America*, 108, 21265–21269. Available from: https://doi.org/10.1073/pnas.1105861108

**Kurita, T., Moroi, K., Iwai, M., Okazaki, K., Shimizu, S., Nomura, S. et al.** (2020) Efficient and multiplexable genome editing using platinum TALENs in oleaginous microalga, *Nannochloropsis oceanica* NIES‐2145. *Genes to Cells*, 25, 695–702. Available from: https://doi.org/10.1111/gtc.12805

**Liu, M., Ding, W., Pan, Y., Hu, H. & Liu, J.** (2023) Zeaxanthin epoxidase is involved in the carotenoid biosynthesis and light‐dependent growth of the marine alga *Nannochloropsis oceanica*. *Biotechnology for Biofuels and Bioproducts*, 16, 1–14. Available from: https://doi.org/10.1186/s13068-023-02326-y

**Liu, M., Ding, W., Yu, L., Shi, Y. & Liu, J.** (2022) Functional characterization of carotenogenic genes provides implications into carotenoid biosynthesis and engineering in the marine alga *Nannochloropsis oceanica*. *Algal Research*, 67, 102853. Available from: https://doi.org/10.1016/j.algal.2022.102853

**Liu, M., Yu, L., Zheng, J., Shao, S., Pan, Y., Hu, H. et al.** (2024) Turning the industrially relevant marine alga *Nannochloropsis* red: one move for multifaceted benefits. *New Phytologist*, 244, 1467–1481. Available from: https://doi.org/10.1111/nph.20114

**Michelberger, T., Mezzadrelli, E., Bellan, A., Perin, G. & Morosinotto, T.** (2025) The xanthophyll cycle balances photoprotection and photosynthetic efficiency in the seawater alga *Nannochloropsis oceanica*. *Plant Physiology*, 198, kiaf301. Available from: https://doi.org/10.1093/plphys/kiaf301

**Moog, D., Stork, S., Reislöhner, S., Grosche, C. & Maier, U.‐G.** (2015) *In vivo* localization studies in the stramenopile alga *Nannochloropsis oceanica*. *Protist*, 166, 161–171. Available from: https://doi.org/10.1016/j.protis.2015.01.003

**Moroi, K., Yamamoto, T. & Kurita, T.** (2025) Double‐strand break‐free and transgene‐free genome editing in the microalga *Nannochloropsis oceanica* using removable vectors containing the CRISPR base editing system. *Scientific Reports*, 15, 1–11. Available from: https://doi.org/10.1038/s41598-025-26657-y

**Naduthodi, M.I.S., Mohanraju, P., Südfeld, C., D'Adamo, S., Barbosa, M.J. & van der Oost, J.** (2019) CRISPR‐Cas ribonucleoprotein mediated homology‐directed repair for efficient targeted genome editing in microalgae *Nannochloropsis oceanica* IMET1. *Biotechnology for Biofuels*, 12, 1–11. Available from: https://doi.org/10.1186/s13068-019-1401-3

**Naduthodi, M.I.S., Südfeld, C., Avitzigiannis, E.K., Trevisan, N., van Lith, E., Alcaide Sancho, J. et al.** (2021) Comprehensive genome engineering toolbox for microalgae *Nannochloropsis oceanica* based on CRISPR‐Cas systems. *ACS Synthetic Biology*, 10, 3369–3378. Available from: https://doi.org/10.1021/acssynbio.1c00329

**Perin, G., Bellan, A., Segalla, A., Meneghesso, A., Alboresi, A. & Morosinotto, T.** (2015) Generation of random mutants to improve light‐use efficiency of *Nannochloropsis gaditana* cultures for biofuel production. *Biotechnology for Biofuels*, 8, 1–13. Available from: https://doi.org/10.1186/s13068-015-0337-5

**Poliner, E., Busch, A.W.U., Newton, L., Kim, Y.U., Clark, R., Gonzalez‐Martinez, S.C. et al.** (2022) Aureochromes maintain polyunsaturated fatty acid content in *Nannochloropsis oceanica*. *Plant Physiology*, 189, 906–921. Available from: https://doi.org/10.1093/plphys/kiac052

**Poliner, E., Clark, E., Cummings, C., Benning, C. & Farre, E.M.** (2020) A high‐capacity gene stacking toolkit for the oleaginous microalga, *Nannochloropsis oceanica* CCMP1779. *Algal Research*, 45, 1–11. Available from: https://doi.org/10.1016/j.algal.2019.101664

**Poliner, E., Cummings, C., Newton, L. & Farré, E.M.** (2019) Identification of circadian rhythms in *Nannochloropsis* species using bioluminescence reporter lines. *The* *Plant Journal*, 99, 112–127. Available from: https://doi.org/10.1111/tpj.14314

**Poliner, E., Takeuchi, T., Du, Z.‐Y., Benning, C. & Farré, E.M.** (2018) Nontransgenic marker‐free gene disruption by an episomal CRISPR system in the oleaginous microalga, *Nannochloropsis oceanica* CCMP1779. *ACS Synthetic Biology*, 7, 962–968. Available from: https://doi.org/10.1021/acssynbio.7b00362

**Poveda‐Huertes, D., Patwari, P., Günther, J., Fabris, M. & Andersen‐Ranberg, J.** (2023) Novel transformation strategies improve efficiency up to 10‐fold in stramenopile algae. *Algal Research*, 74, 1–11. Available from: https://doi.org/10.1016/j.algal.2023.103165

**Robinson, K., Chia, K.‐S., Guyon, A., Schornack, S. & Carella, P.** (2024) An efficient sulfadiazine selection scheme for stable transformation in the model liverwort *Marchantia polymorpha*. *Journal of Experimental Botany*, 75, 5585–5591. Available from: https://doi.org/10.1093/jxb/erae256

**Roth, M.S., Cokus, S.J., Gallaher, S.D., Walter, A., Lopez, D., Erickson, E. et al.** (2017) Chromosome‐level genome assembly and transcriptome of the green alga *Chromochloris zofingiensis* illuminates astaxanthin production. *Proceedings of the National Academy of Sciences of the United States of America*, 114, E4296–E4305. Available from: https://doi.org/10.1073/pnas.1619928114

**Südfeld, C., Hubáček, M., Figueiredo, D., Naduthodi, M.I.S., van der Oost, J., Wijffels, R.H. et al.** (2021) High‐throughput insertional mutagenesis reveals novel targets for enhancing lipid accumulation in *Nannochloropsis oceanica*. *Metabolic Engineering*, 66, 239–258. Available from: https://doi.org/10.1016/j.ymben.2021.04.012

**Südfeld, C., Kiyani, A., Buckens, H., Hubáček, M., Wijffels, R.H., Barbosa, M.J. and D'Adamo, S.** (2022a) Accumulation of medium chain fatty acids in *Nannochloropsis oceanica* by heterologous expression of *Cuphea palustris* thioesterase FatB1. *Algal Research*, 64, 1–12. Available from: https://doi.org/10.1016/j.algal.2022.102665

**Südfeld, C., Kiyani, A., Wefelmeier, K., Wijffels, R.H., Barbosa, M.J. & D'Adamo, S.** (2023) Expression of glycerol‐3‐phosphate acyltransferase increases non‐polar lipid accumulation in *Nannochloropsis oceanica*. *Microbial Cell Factories*, 22, 1–14. Available from: https://doi.org/10.1186/s12934-022-01987-y

**Südfeld, C., Pozo‐Rodríguez, A., Manjavacas Díez, S.A., Wijffels, R.H., Barbosa, M.J. & D'Adamo, S.** (2022b) The nucleolus as a genomic safe harbor for strong gene expression in *Nannochloropsis oceanica*. *Molecular Plant*, 15, 340–353. Available from: https://doi.org/10.1016/j.molp.2021.11.003

**Verruto, J., Francis, K., Wang, Y., Low, M.C., Greiner, J., Tacke, S. et al.** (2018) Unrestrained markerless trait stacking in *Nannochloropsis gaditana* through combined genome editing and marker recycling technologies. *Proceedings of the National Academy of Sciences of the United States of America*, 115, E7015–E7022. Available from: https://doi.org/10.1073/pnas.1718193115

**Vieler, A., Wu, G., Tsai, C.‐H., Bullard, B., Cornish, A.J., Harvey, C. et al.** (2012) Genome, functional gene annotation, and nuclear transformation of the heterokont oleaginous alga *Nannochloropsis oceanica* CCMP1779. *PLoS Genetics*, 8, e1003064. Available from: https://doi.org/10.1371/journal.pgen.1003064

**Vogler, B.W., Ashford, A. & Posewitz, M.C.** (2021) CRISPR/Cas9 disruption of glucan synthase in *Nannochloropsis gaditana* attenuates accumulation of *β*‐1,3‐glucose oligomers. *Algal Research*, 58, 1–7. Available from: https://doi.org/10.1016/j.algal.2021.102385

**Wang, Q., Lu, Y., Xin, Y., Wei, L., Huang, S. & Xu, J.** (2016) Genome editing of model oleaginous microalgae *Nannochloropsis* spp. by CRISPR/Cas9. *The Plant Journal*, 88, 1071–1081. Available from: https://doi.org/10.1111/tpj.13307

**Zhu, H., Wu, Y., Feng, M., Ouyang, L., Su, H. & Wei, L.** (2025) Epitranscriptomic reprogramming in response to low CO_2_ stress and m^6^A engineering to enhance biomass production in *Nannochloropsis oceanica*. *The* *Plant Journal*, 124, e70566. Available from: https://doi.org/10.1111/tpj.70566
